# The Nuclear Effector RIRG190 Interacts with SAS10 to Regulate Arbuscular Mycorrhizal Symbiosis

**DOI:** 10.3390/ijms262412178

**Published:** 2025-12-18

**Authors:** María Victoria Aparicio Chacón, Annick De Keyser, Naomi Stuer, Toon Leroy, Evi Ceulemans, Juan Antonio López-Ráez, Alain Goossens, Judith Van Dingenen, Sofie Goormachtig

**Affiliations:** 1 Department of Plant Biotechnology and Bioinformatics, Ghent University, 9052 Gent, Belgium; 2 Center for Plant Systems Biology, VIB, 9052 Gent, Belgium; 3 Department of Soil and Plant Microbiology, Estación Experimental del Zaidín (EEZ-CSIC), 18008 Granada, Spain

**Keywords:** *Rhizophagus irregularis*, AM symbiosis, effector protein, *Solanum lycopersicum*, *Arabidopsis thaliana*, rRNA biogenesis, nucleus

## Abstract

Most land plants engage in a mutualistic interaction with arbuscular mycorrhizal fungi (AMF), for which *Rhizophagus irregularis* is a model species. Like plant pathogenic fungi, AMF genomes encode hundreds of putative effector proteins. However, for only a few, the molecular mechanisms by which they alter the host’s physiology are known. Here, we combined several reverse genetic approaches to unravel the role of the RIRG190 effector protein in arbuscular mycorrhiza (AM) symbiosis. Using multiple heterologous tools, evidence is provided that the RIRG190 effector is secreted and localizes to the plant nucleus. Moreover, by means of yeast two-hybrid (Y2H) and ratiometric bimolecular fluorescence complementation (rBIFC) assays, the data demonstrate that RIRG190 interacts with the protein Something About Silencing (SAS10), known to be involved in rRNA biogenesis in the nucleolus of cortical cells. Our findings suggest that rRNA biogenesis is a key process modulated by AMF, potentially to enhance plant metabolic activity, facilitating cell cycle progression, and to support the establishment of the symbiosis.

## 1. Introduction

Arbuscular mycorrhizal fungi (AMF) are root symbionts that help approximately two-thirds of the land plants to overcome nutrient deficiency, while improving tolerance to a changing environment [1,2,3,4,5]. The successful establishment of the AM symbiosis requires a complex and fine-tuned exchange of communication signals between both partners, with AM symbionts undergoing a drastic reprogramming of the cellular homeostasis and a shift in the root gene expression profile to promote fungal colonization [6,7]. Forward and reverse genetic studies in AM host plants have revealed the importance of the so-called symbiosis (SYM) pathway, responsible for decoding fungal Myc-factor signatures and for orchestrating the downstream cellular events essential for fungal colonization [8,9,10,11]. Alongside the SYM pathway, cellular changes, such as cytoskeleton redistribution, vacuole fragmentation, activation of ion transport, plant nuclear movements, and cell cycle modulation, are promoted to allow fungal niche occupation [12,13,14,15,16]. Once the pre-symbiotic phase has been set, the fungus grows through the different cell layers until reaching the root inner cortex, where its hyphae branch intracellularly to develop into arbuscules [6]. These arbusculated cells represent major functional units in which nutrients, and probably other molecules, are traded between both organisms [17,18,19,20,21,22,23]. Yet, most of the fungal cues that participate in AM symbiosis beyond the pre-symbiotic phase remain to be characterized.

During plant–microbe interactions, microorganisms secrete and can further translocate proteinaceous effectors inside the host plant cells [24,25,26,27]. Once internalized, effectors bind and adjust the activity of host proteins, DNA, and/or RNA to influence the host physiology to ease microbial colonization [28,29,30]. Equally, AMF make use of such strategies to regulate the mycorrhization outcome [31,32,33,34]. Yet, effective elucidation of the role of AMF effector proteins is hampered by the lack of functionally characterized protein domains in their sequences and the impossibility of genetically modifying AMF [35,36]. For these reasons, the identification of the effector’s plant target(s) is an important aspect to infer effector function. Despite the fact that several hundred transcriptionally active putative *Rhizophagus irregularis* (*Rhizophagus*) effectors have been identified in different plant host species, only few have been investigated for their function in AM symbiosis, with a strong focus on the model plant *Medicago truncatula* (Medicago) [37,38,39,40,41,42]. Remarkably, a detailed understanding of the molecular mechanisms employed by *Rhizophagus* intracellular effectors to influence the plant host physiology is just starting to increase [37,41]. The *Rhizophagus* nuclear-localized effector1 (RiNLE1) and secreted protein 7 (SP7) effectors regulate plant host immunity by binding to histone 2B (H2B) or by interacting with the pathogenesis-related transcription factor Ethylene response factor 19 (ERF19), respectively [37,41]. Further investigation of the SP7-like effector family demonstrated association of several effectors with the serine/arginine (SR)-rich protein 45 to interfere with alternative splicing of immunity-related genes [43]. Finally, the nuclear-localized effectors GLOIN707, GLOIN781, GLOIN261, and RiSP749 might tackle different molecular processes, such as DNA replication, methylglyoxal detoxification, and RNA splicing, through association with specific host plant nuclear targets [42]. Thus, while vast improvements have been made in the AMF effector field in the last decade, comprehensive knowledge of the molecular processes exploited by fungal effectors during AM symbiosis is still scarce.

During microbial establishment, the transcriptional and translational machinery of host root cells gets boosted to activate many key functional processes to allow infection and accommodate the microbe. An example is the induction of changes in the host cell cycle to allow colonization, also occurring during legume nodulation or pathogenic root-knot nematode infestation [15,44,45,46]. Activation of ectopic cortical cell division prior to AMF colonization, as well as a rise in ploidy levels and increased nuclear sizes during fungal accommodation, have been reported [15,16,47,48,49]. Hence, the fine-tuning of the host cell molecular machinery and metabolism is essential for fungal progression and niche occupation in AM symbiosis.

In this work, we investigated the role of the *Rhizophagus* effector protein RIRG190 in AM symbiosis following the experimental workflow schematically summarized in Figure S1. *RIRG190* is expressed in mycorrhized *Solanum lycopersicum* (tomato) roots, has a functional signal peptide (SP) for its secretion, carries a predicted nuclear localization signal (NLS), and is mainly localized in the plant nucleus, with enhanced accumulation in nucleolar-like structures. Ectopic expression of *RIRG190* in tomato positively influenced arbuscule abundance. We identified the host plant’s SlSAS10 nuclear protein as an RIRG190-interacting protein and, by means of reverse genetic studies and functional analysis, we hypothesize that the protein complex formed by RIRG190 and SlSAS10 can adjust rRNA biogenesis to guarantee a balanced AM symbiosis.

## 2. Results

### 2.1. RIRG190 Is a Potential Nuclear Effector Protein Secreted During AM Symbiosis in Tomato

**Figure 1:**
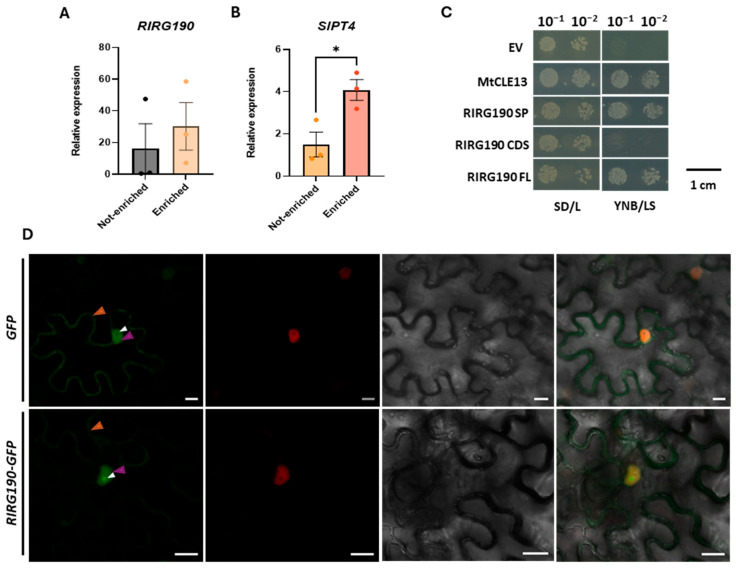
RIRG190 is a nuclear effector protein potentially secreted during AM symbiosis in tomato (**A**,**B**) *RIRG190* (**A**) and *SlPT4* (**B**) gene expression levels in *SlPT4p:GFP RolDp:mRuby-NLS* mycorrhized tomato roots at 2 wpi. *RIRG190* values were normalized using *RiEF1α* and *SlPT4* values using *SlEF1α* and *SlGAPDH* housekeeping genes and the arbuscule-enriched regions were relatively compared to non-enriched samples. Data are represented as mean ± SEM of two independent biological repeats (n = 12; * *p* < 0.05; Student’s *t*-test). (**C**) YST experiment performed with the three different RIRG190 effector sequence parts, i.e., the signal peptide (SP), the coding sequence without SP (CDS), and the full length (FL), fused to the *SUC2* gene in the pYST1 vector. As a negative control for secretion, the empty vector (EV) was used, while MtCLE13 was used as a positive control. Positively transformed Y02321 colonies were diluted and grown on SD/L control growth medium and on YNB/LS sucrose selective medium for 3 days at 30 °C. (**D**) Subcellular localization of the *RIRG190-GFP* fusion protein. *35Sp:GFP RolDp:mRuby-NLS* (upper row) and *35Sp:RIRG190-GFP RolDp:mRuby-NLS* (lower row) were transiently overexpressed in tobacco leaf cells. Arrowheads indicate the cytoplasm (orange), nucleus (purple), and nucleolus (white). The left panel is the GFP signal, the second panel is the mRUBY fluorescence signal, the third panel is the bright field, and the right panel is a merge between the three. A minimum of three individual tobacco leaves were infiltrated and analyzed, all showing the same localization. Bars, 20 µM.

RIRG190 was formerly annotated as a potential effector protein from *R. irregularis* [34] that contains a “cysteine-rich secretory proteins, antigen 5, and pathogenesis-related 1 (PR1) proteins” (CAP) domain, also found in other effectors from the pathogenic fungus *Ustilago maydis* [50]. RIRG190 is a small protein that carries a putative N-terminal SP, which could allow its secretion outside the fungal cell [51] as well as a predicted NLS for its possible translocation into the plant nucleus (Figure S2A) [51,52]. *RIRG190* has been shown to be expressed in many mycorrhized hosts [34], but expression data was lacking in tomato. To get insight into *RIRG190* expression during AM symbiosis in tomato, tomato composite plants expressing *SlPT4p:GFP RolDp:mRuby-NLS* were generated as previously described [42,53,54]. These composite plants display a wild-type (WT) shoot and transgenic roots, with each hairy root resulting from independent transformation events [53]. While nuclear mRuby fluorescence indicates positively transformed roots, green fluorescent protein (GFP) driven by the arbuscule-specific promoter *SlPT4* reports the presence of arbuscule-containing cells (Figure S2B) [54,55]. We investigated the *RIRG190* expression levels in GFP-enriched regions and compared them to those in regions depleted of arbusculated cells at two weeks post-inoculation (wpi). When normalized against the fungal gene *RiEF1α*, *RIRG190* gene expression was detected in both root sections (Figure 1A), suggesting a broad expression of *RIRG190* inside the fungus throughout the different stages of the symbiotic process. The arbuscule-specific phosphate transporter-encoding gene *SlPT4* [53] was significantly upregulated in the arbuscule-enriched fraction compared to the non-enriched material at 2 wpi (Figure 1B), confirming functional symbiosis at this timepoint.

Because RIRG190 carries a putative N-terminal SP (Figure S2A) [51], its secretion ability was tested through the yeast secretion trap (YST) assay [56]. To this end, the predicted RIRG190 SP, the effector coding sequence (CDS) lacking the SP, or the effector full length (FL) were fused to the sucrose invertase (*SUC*) gene and transformed into a *SUC*-deficient *Saccharomyces cerevisiae* (yeast) strain [57]. Only yeasts expressing *SUC2* fusions with a functional SP for extracellular secretion can metabolize the sucrose in the medium to support development. Growth was detected for all yeasts in the control medium, whereas in the sucrose-supplemented medium, yeast growth was only observed for those expressing the *RIRG190 SP*- and *FL-SUC2* fusions, indicating that the RIRG190 SP is functional (Figure 1C).

Because the in silico analysis demonstrated a putative NLS site in the C-terminal region of the RIRG190 protein (Figure S2A) [52], we investigated whether the effector localizes in plant nuclei by transiently overexpressing *RIRG190-GFP* in *Nicotiana benthamiana* (tobacco) leaves. Fluorescence was detected both in the cytosol and in the nucleus, with a strong GFP signal in nucleolar-like structures in *RIRG190-GFP*-overexpressing cells that was absent in the nucleus of cells overexpressing *GFP* (Figure 1D). Western blot analysis confirmed the integrity of the fusion protein in the raw protein extract from infiltrated tobacco leaves (Figure S2C).

Additionally, we investigated whether homologous RIRG190 effector-like proteins were present in other organisms, as described by Wang et al. [41] based on sequence similarity. Interestingly, RIRG190-like effectors were only found in AMF species, including *Rhizophagus* sp., *Funneliformis* sp., *Gigaspora* sp., as well as *Cetraspora pellucida* and *Ambispora gerdemannii*, suggesting their involvement in AM-dependent processes (Figure S2D). However, it was shown that certain structurally similar effector families are conserved across unrelated microbes, even when their sequences are not closely related [58,59,60]. Therefore, a structural similarity search was performed using Foldseek in AlphaFold, revealing multiple structurally similar SCP/CAP domain-containing proteins with low sequence identity in both bacterial and fungal species (Figure S2E).

Taken together, RIRG190 fulfills all the necessary criteria to be considered as a potentially secreted and nuclear-localized effector conserved among AMF.

### 2.2. Ectopic RIRG190 Expression Impacts the Morphology of Arbusculated Cells

To gain insights into the role of RIRG190 in AM symbiosis, tomato composite plants ectopically overexpressing *RIRG190-GFP* were generated. The expression of the correct fusion protein was confirmed by Western blot (Figure S3B) and RT-qPCR (Figure S3C). Confocal microscopy showed that RIRG190-GFP was also localized in the cytosol and nucleus of mycorrhized tomato root cells, especially in nucleolar-like structures (Figure S3A).

**Figure 2:**
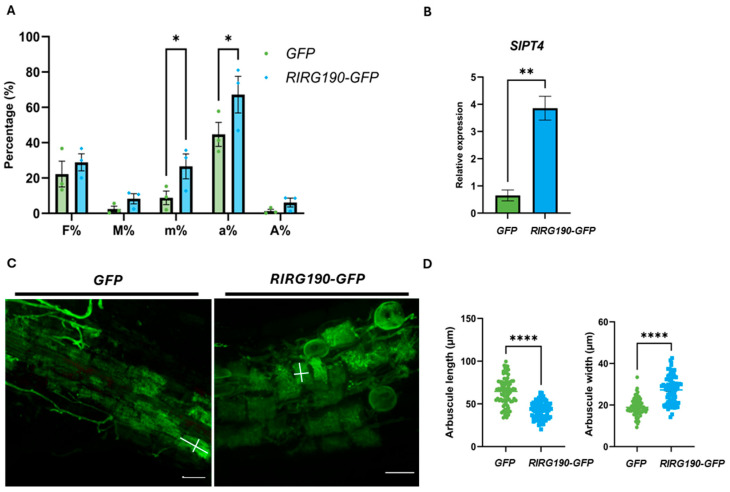
RIRG190 affects the morphology of cells containing arbuscules and increases mycorrhization. (**A**) Mycorrhization levels of tomato composite roots expressing *GFP* or *RIRG190-GFP* at 4 wpi according to the Trouvelot method. F%, mycorrhization frequency in the root; M%, mycorrhization intensity in the root; m%, mycorrhization intensity in mycorrhizal parts of the root fragments; a%, arbuscule abundance in mycorrhizal parts of the root fragments; A%, arbuscule abundance in the root. Values are means of three independent biological repeats with their SEM (n = 12; *, *p* < 0.05; Two-way ANOVA followed by multiple comparisons, α < 0.05). (**B**) Gene expression levels of tomato *SlPT4* in mycorrhized root cells expressing *GFP* or *RIRG190-GFP*. Tomato gene normalization was conducted using *SlEF1α* and *SlGAPDH* and relatively compared to mycorrhized *GFP* control roots at 4 wpi. Data are means ± SEM of three independent biological repeats (n = 9–12; **, *p* < 0.005; Student’s *t*-test). (**C**) Wheat germ agglutinin (WGA)-stained arbuscule-containing regions of tomato roots expressing *GFP* or *RIRG190-GFP*. The white lines indicate the arbuscule width and length of the arbuscule-containing cells. Bars, 50 µM. (**D**) Quantification of length and width of WGA-stained arbuscules in mycorrhized tomato root cells expressing *GFP* or *RIRG190-GFP*. Data are means of 80–81 individual quantifications with their SEM (****, *p* < 0.0001; One-way ANOVA).

Next, mycorrhization levels were quantified at 4 wpi following the Trouvelot method [61]. A significant increase in relative arbuscule abundance (a%) and mycorrhization intensity (m%) in the analyzed root fragments was observed in *RIRG190-GFP* tomato roots when compared to the *GFP* plants (Figure 2A), while no significant differences could be detected for the mycorrhization frequency and intensity, or the arbuscule abundance in the whole root system (Figure 2A, F%, M% and A%, respectively). Also, higher transcript levels of *SlPT4* were detected when compared to mycorrhized *GFP* control plants (Figure 2B). Furthermore, ink-colored *RIRG190-GFP* arbusculated cells looked smaller and square-shaped, and clustered more closely together compared to those of *GFP* control plants (Figure S3D). To gain more insights into the size of arbuscule-containing cells, a morphological analysis was conducted on wheat germ agglutinin (WGA)-fluorescent-stained arbuscules as described by Voß et al. [39]. Arbusculated cells overexpressing *RIRG190-GFP* displayed a significant decrease in length compared to those of *GFP* control plants (Figure 2C,D), while the width of arbuscule-containing cells was significantly increased (Figure 2C,D).

To conclude, ectopic *RIRG190-GFP* expression positively affects symbiosis and influences the architecture of arbuscule-containing cortical cells.

### 2.3. RIRG190 May Play a Role in Promoting Cortical Cell Division in Arabidopsis

**Figure 3:**
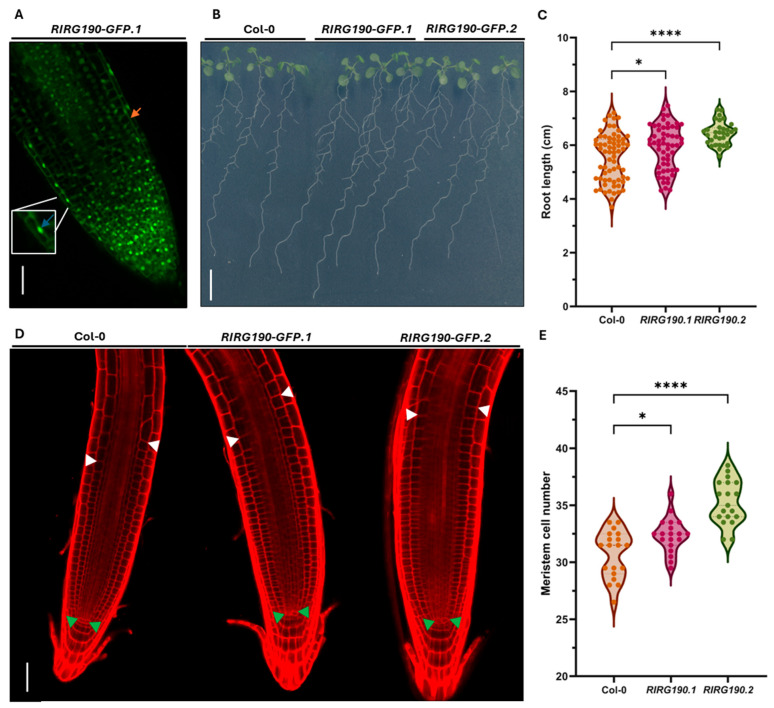
RIRG190 plays a positive role in primary root length growth by increasing the number of meristematic cortical cells in a non-host plant. (**A**) Representative confocal imaging of an *RIRG190-GFP* Arabidopsis transgenic root tip. The fluorescent signal detected in the nucleus is marked with a blue arrow (inset), and the cytoplasmic signal is indicated by an orange arrow. Bar, 20 µM. (**B**) Root phenotype of Arabidopsis Col-*0* and the two *RIRG190-GFP* lines at 14 days after stratification (DAS). Bar, 1 cm. (**C**) Primary root length of Arabidopsis Col-*0*, *RIRG190-GFP.1*, and *RIRG190-GFP.2* measured at 14 DAS. Values are means of two biological repeats (n = 32–66; *, *p* < 0.05; ****, *p* < 0.0001; One-way ANOVA). (**D**) Confocal laser-scanning microscopy pictures of 6-day-old Arabidopsis root tips of Col-*0*, *RIRG190-GFP.1*, and *RIRG190-GFP.2* incubated with 1% propidium iodide. Green arrowheads indicate the end of the quiescent center, white arrowheads mark the first elongated cortical cell of the root differentiation zone. Bar, 50 µM. (**E**) Number of meristematic cortical cells in the root apical meristem of Arabidopsis Col-*0* and the two *RIRG190-GFP* lines at 6 DAS. Values are means of three independent biological repeats (n = 19; *, *p* < 0.05; ****, *p* < 0.0001; One-way ANOVA).

The ectopic expression of *RIRG190* impacts the morphology of arbuscule-containing cortical cells in tomato, suggesting its possible implication in cell patterning-related processes such as cell division. Composite plants display chimeric gene expression, challenging the identification of subtle plant phenotypical traits. To decipher this influence on cortical cell morphology and whether it is conserved in distantly related plants unable to engage in AM symbiosis, two Arabidopsis homozygous transgenic lines overexpressing *RIRG190-GFP* (*RIRG190-GFP.1* and *RIRG190-GFP.2*) were generated. After confirming the predominantly nuclear localization of *RIRG190-GFP* in these roots (Figure 3A), the gene overexpression levels and the protein fusion integrity (Figure S4A,B), the primary root length was analyzed at 14 days (Figure 3B). The root length was significantly increased in both lines when compared to Colombia-*0* (Col-*0*) WT plants (Figure 3C), while lateral root density was not affected (Figure S4C) [42]. Average root length was 5.53 ± 0.89 in Col-0, 5.91 ± 0.82 in *RIRG190-GFP.1*, and 6.44 ± 0.42 in *RIRG190-GFP.2.* Compared to Col-0, *RIRG190-GFP.1* exhibited a mean increase of 0.38 (1.07-fold), while *RIRG190-GFP.2* showed a mean increase of 0.91 (1.16-fold). Next, to gain insights into the underlying cellular process responsible for the longer *RIRG190-GFP* primary roots, the number of cortical cells present in the root apical meristem was determined in six-day-old seedlings (Figure 3D). Cortical cell number was 30.7 ± 2.0 in Col-0, 32.4 ± 1.5 in *RIRG190-GFP.1*, and 35.3 ± 1.9 in *RIRG190-GFP.2*. Compared to Col-0, *RIRG190-GFP.1* exhibited a mean increase of 1.66 (1.05-fold), while *RIRG190-GFP.2* showed a mean increase of 4.55 (1.15-fold) (Figure 3E) [62].

Taken together, these results show that the ectopic expression of *RIRG190-GFP* is capable of promoting root growth by increasing cell division in the Arabidopsis root apical meristem, and that this process does not require the participation of any other fungal molecule.

### 2.4. The RIRG190 Effector Interacts with the Tomato Protein SAS10 in the Plant Nucleus

**Figure 4:**
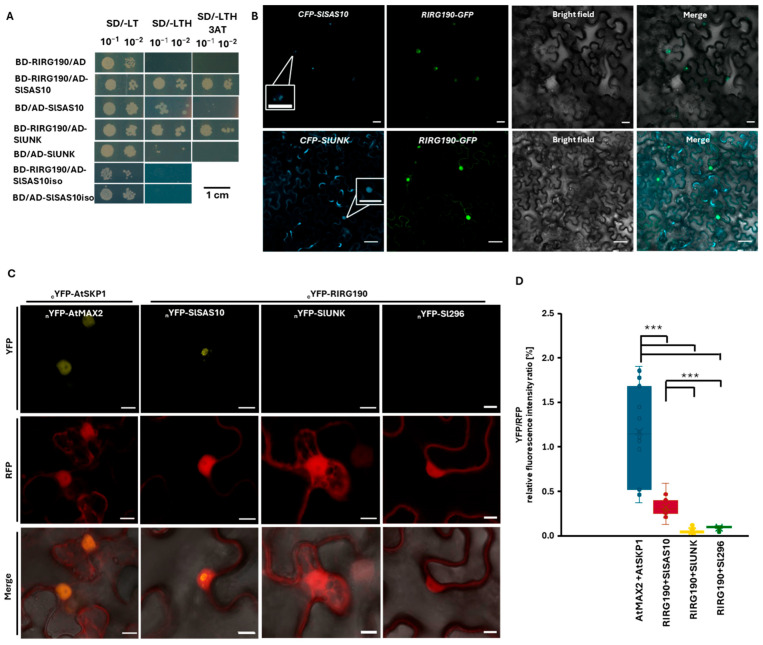
RIRG190 strongly interacts with SlSAS10 in the plant nucleus. (**A**) Binary Y2H assays between RIRG190 and SlSAS10 and SlUNK proteins on selective medium, and the tomato SlSAS10 isoform (SlSAS10iso). As a negative control, the tomato baits were cotransformed with the empty PGBKT7 vector (BD/+). Transformed PJ-69α cells were diluted and grown on SD/-LT control medium and SD/-LTH selective medium with or without 5 mM of 3-AT for 3 days at 30 °C. (**B**) Confocal laser-scanning microscopy images of N-terminal *CFP-SlSAS10* and *CFP-SlUNK* and C-terminal *RIRG190-GFP* fusions in tobacco leaf epidermal cells. CFP fluorescent signals of the SlUNK fusion protein were detected in the cytoplasm and nuclei; those of the *CFP-SlSAS10* fusion were restricted to nuclear foci (insets). Merged fluorescent GFP-CFP signals indicate nuclear colocalization of both tomato proteins with the RIRG190 effector. Bars, 10 µM. (**C**) rBiFC assay of RIRG190 and SlSAS10 N-terminal split YFP fusions. AtSKP1-AtMAX2 protein pair was used as a positive control, RIRG190-SlUNK and RIRG190-Sl296 protein fusion pairs were used as negative controls. The RFP fluorescent signal corresponds to the constitutively expressed control cassette (middle pictures). Bottom pictures show the overlay between YFP/RFP fluorescence. Three independent experiments were conducted, and a total of 18–26 cells were analyzed. Bars, 10 µM. (**D**) YFP/RFP relative fluorescent intensity analysis of the rBiFC protein pairs AtSKP1-AtMAX2 (positive control), RIRG190-SlSAS10, RIRG190-SlUNK, and RIRG190/Sl296. Data are shown as means of three biological replicates ± SEM (n = 18–26; ***, *p* < 0.0001; Two-way ANOVA analysis followed by multiple comparison (α < 0.05)).

We hypothesized that, similarly to other microbial effector molecules, RIRG190 might interact with a plant protein to exert its function [30,63,64]. Therefore, to identify a possible RIRG190 plant target, a yeast two-hybrid (Y2H) screening was performed against a tomato root cDNA library using RIRG190 as bait. Only four candidates were identified (Dataset S1A). Y2H pairwise verifications confirmed only two interacting tomato proteins, SlSAS10 (Solyc11g072390) and SlUNK (Solyc07g045450; Figure 4A). Because SlSAS10 has two isoforms, a Y2H screening was independently conducted using the additional SlSAS10 isoform (Solyc11g072320) as prey, demonstrating that RIRG190 specifically interacts with SlSAS10 (Solyc11g072390) (Figure 4A).

Next, an in silico study was conducted using InterProScan to identify specific protein domains in the two interacting proteins. While no features were detected in the SlUNK protein, two NLS sites as well as two functionally characterized SAS10/Utp3/C1D protein domains were identified in the SlSAS10 protein sequence (Figure S5A). The SAS10/Utp3/C1D domain is described to act as a scaffold domain, providing support for proteins interacting with ribonucleic acids to modulate chromatin silencing, rRNA processing, RNA surveillance, and DNA repair [65,66,67,68,69,70]. Hence, altogether, SlSAS10 might localize in the plant nucleus, where it could be exerting a key role in several essential molecular pathways required for the establishment of AM.

As the data demonstrated that RIRG190 predominantly localizes in the plant nucleus, we expected SlSAS10 and SlUNK to localize in the same subcellular compartment. Therefore, both N-terminally cyan fluorescent protein (CFP) fusions were transiently overexpressed in tobacco leaves. Fluorescent signals from SlUNK accumulated in the cytoplasm and were weakly visible in the plant nucleus (Figure S5B). In contrast, SlSAS10 fluorescence was restricted to one or two nuclear condensates, possibly corresponding to the nucleolus (Figure S5B). Merging the confocal images of RIRG190-GFP with those of SlSAS10-CFP and SlUNK-CFP confirmed their common nuclear localization (Figure 4B).

To detect whether and where RIRG190 interacts with both tomato proteins, a ratiometric bimolecular fluorescence complementation (rBiFC) assay was performed in tobacco leaves. This technique not only allows the detection of the interaction after yellow fluorescent protein (YFP) reconstitution, but it also guarantees an equal gene dosage and facilitates the ratiometric quantification between fluorescence ratios from different protein pairs, thanks to the constitutively expressed red fluorescent protein (RFP) cassette [71]. As a positive control, the Arabidopsis nuclear-localized AtSKP1 and AtMAX2 proteins were N-terminally fused to their corresponding YFP halves [72]. As a negative control, the interaction of RIRG190 was tested with Sl296, a tomato protein that was previously demonstrated by rBiFC to strongly interact in the nucleolus with the nuclear effector GLOIN707 [42,72]. No interaction was detected for the RIRG190-SlUNK and RIRG190-Sl296 combinations, whereas a reconstituted YFP was visualized for the AtSKP1-AtMAX2 positive control and the RIRG190-SlSAS10 pair (Figure 4C and Figure S5C). Additionally, nuclear YFP/RFP relative fluorescent intensity ratios were significantly different between the positive control and the RIRG190-SlSAS10 combination with the RIRG190-SlUNK and RIRG190-Sl296 combinations (Figure 4D), confirming the specific in vivo physical association of RIRG190-SlSAS10 (Figure 4C). To further test the specificity of SlSAS10 with the RIRG190 effector, its interaction was tested with GLOIN707, a previously published *R. irregularis* effector suggested to also act in the nucleolus, via Y2H and rBIFC analysis [42]. No growth was observed on SD-LTH for this interaction (Figure S6A), and YFP was not reassembled for the GLOIN707-SlSAS10 and GLOIN707-SlUNK combinations (Figure S6B,C). Thus, we can conclude that the nuclear proteins RIRG190 and SlSAS10 interact in planta.

### 2.5. Mis-Regulation of SlSAS10 Expression Causes Impaired Mycorrhization

To understand the potential role of SlSAS10 in arbuscular mycorrhization, the *SlSAS10* gene expression levels were analyzed in mycorrhized tomato *SlPT4:GFP* root material, as previously described [42,53,54]. *SlSAS10* transcripts were not changed at 2 wpi and significantly downregulated at 4 wpi in both arbuscule-enriched and non-enriched regions compared to the mock sample (Figure S7A).

To decipher the precise tissue in which *SlSAS10* was transcriptionally active, we investigated the β-glucuronidase (GUS) enzymatic activity in tomato composite plants transformed with the *SlSAS10p:GUS* construct at 4 wpi. In non-inoculated roots, the *GUS* signal was observed in the root vasculature (Figure S7B) and in the root apical meristem (Figure S7C). Also in *35S:GUS* lines, we observed a GUS signal in root vasculature and root apical meristem, while in empty vector-transformed roots, no GUS signal was detected (Figure S7G). In the presence of *Rhizophagus*, *GUS* activity was seen in specific cells, some of which displayed higher *GUS* expression than others (Figure S7D). To determine whether those *GUS*-enriched cells might host fungal structures, we conducted a WGA fluorescent costaining that confirmed the overlay between cortical cells containing a strong *GUS* signal (Figure S7E) and WGA-stained arbuscules (Figure S7F).

**Figure 5:**
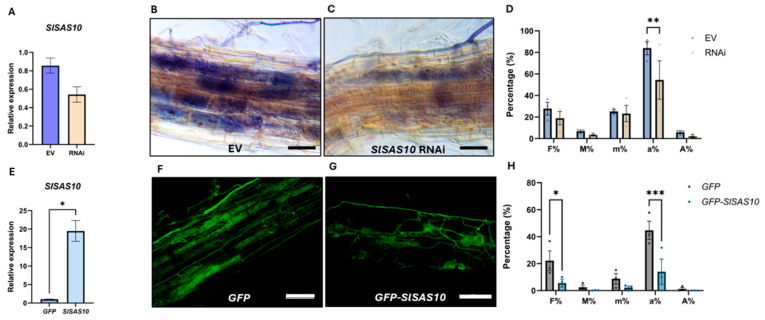
SlSAS10 and RIRG190 participate in the arbuscular phase of AM symbiosis. (**A**,**E**) *SlSAS10* transcript levels in *SlSAS10* RNAi roots (**A**) and in *GFP-SlSAS10* 4-week-old mycorrhized composite plants (**E**), with their respective EV and *GFP* controls. Values were normalized using *SlEF1α* and *SlGAPDH* housekeeping genes and relatively compared to inoculated control roots. Data are shown as means of two and three biological replicates ± SEM (n = 4–6; *, *p* < 0.05; Student’s *t*-test), respectively. (**B**,**C**) Ink-colored root sections of mycorrhized tomato plants expressing the RNAi empty vector (EV) (**B**) or the *SlSAS10* RNAi vector (**C**). Bars, 50 µM. (**D**,**H**) Trouvelot AM quantification of tomato *SlSAS10* RNAi roots (**D**) and *GFP-SlSAS10* roots (**H**), with their respective EV and *GFP* controls at 4 wpi. Data are shown as means of two biological replicates ± SEM (n = 4–6; **, *p* < 0.005; Two-way ANOVA followed by multiple comparison (α < 0.05)) and three biological replicates ± SEM (n = 12; *, *p* < 0.05; ***, *p* < 0.001; Two-way ANOVA followed by multiple comparison (α < 0.05)), respectively. (**F**,**G**) WGA-stained arbuscule-containing regions of tomato roots expressing *GFP* (**F**) or *GFP-SlSAS10* (**G**). Bars, 100 µM.

To further reveal whether and in which phase SlSAS10 could play a role in AM symbiosis, we assessed the effect of *SlSAS10* knockdown in mycorrhized composite plants. As a control, tomato plants carrying the empty hairpin vector (RNAi EV) were used. After verification of the decreased transcriptional levels of *SlSAS10* in the RNAi composite plants (Figure 5A), we conducted an ink root staining (Figure 5B,C) followed by a Trouvelot scoring that evidenced a significant decrease in arbuscule abundance within the root fragments (a%) in *SlSAS10* RNAi compared to the EV control roots, while no changes in other parameters were observed (Figure 5D). Hence, partial downregulation of *SlSAS10* negatively affects arbuscule formation in cortical cells.

We then hypothesized that overexpression of *SlSAS10* might lead to a gain of function in mycorrhization performance. To test this hypothesis, tomato composite plants expressing *35Sp:GFP* or *35Sp:GFP-SlSAS10* were subjected to mycorrhization for 4 weeks. After confirming the upregulation of *SlSAS10* transcript levels in the studied roots (Figure 5E), WGA-stained transgenic roots (Figure 5F,G) were scored in agreement with the Trouvelot method [61]. Contrary to our expectations, *GFP*-*SlSAS10* overexpression led to a significant reduction in arbuscule abundance (a%) and mycorrhization frequency (F%) when compared with mycorrhized *GFP* control roots (Figure 5H).

As a result, we can conclude that *SlSAS10* expression needs to be tightly controlled to guarantee appropriate arbuscule establishment and development in tomato roots.

### 2.6. RIR190 Forms a Protein Complex with Known SAS10 Protein Complex Components in Tomato and Arabidopsis

**Figure 6:**
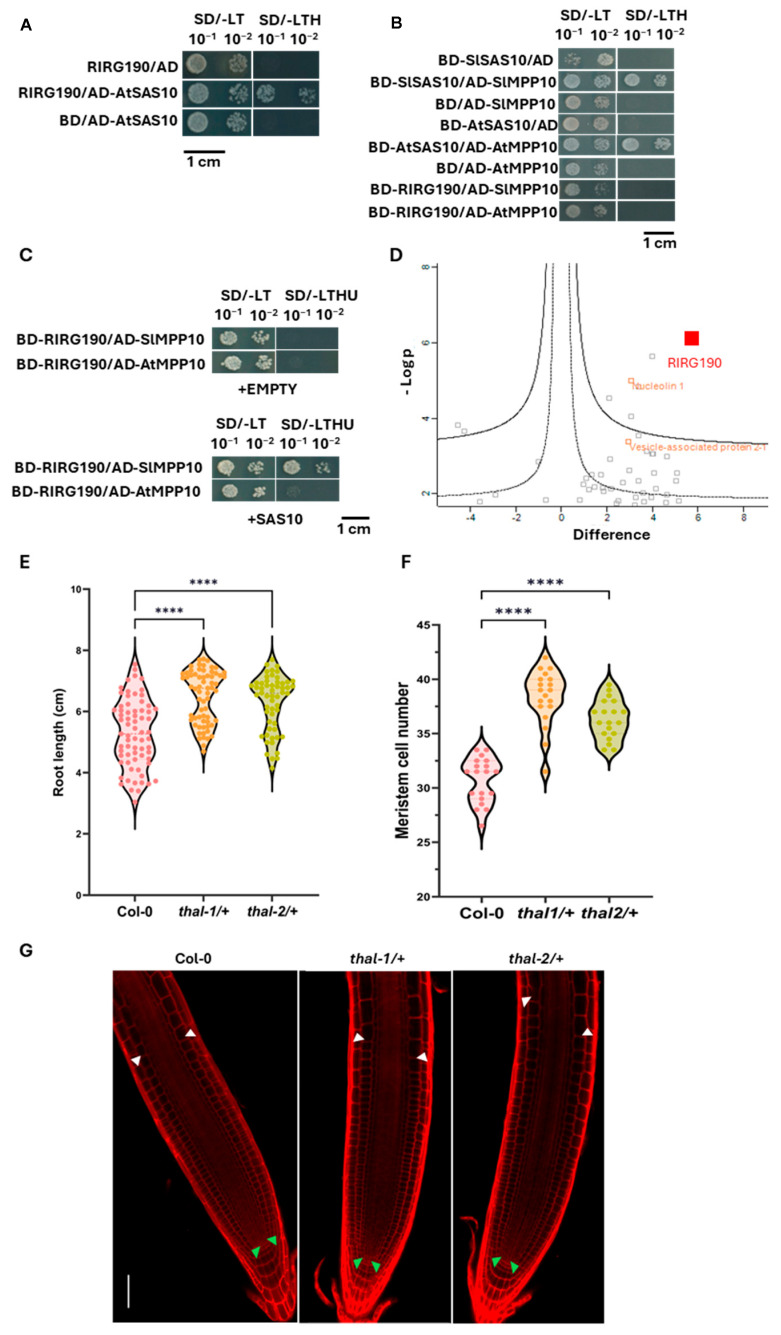
RIRG190-SAS10 interplay is conserved in the non-AM host Arabidopsis. (**A**) Y2H assay between RIRG190 and the Arabidopsis AtSAS10. SAS10 prey proteins were cotransformed with the empty PGBKT7 bait vector or the RIRG190 bait. Transformed PJ-69α yeast cells were diluted and grown in SD/-LT control medium and SD/-LTH selective medium for 3 days at 30 °C. (**B**) Y2H assay between SAS10 and MPP10 in Arabidopsis and tomato, and between RIRG190 and all the MPP10 proteins from both plant species. Homologous protein prey were cotransformed with the empty PGBKT7 bait vector. Transformed yeasts were diluted and grown in SD/-LT control medium and SD/-LTH selective medium for 3 days at 30 °C. (**C**) Y3H between RIRG190/SAS10/MPP10 protein complex. Upper panel, no SAS10 co-transformation. Lower panel, SlSAS10 was co-transformed. Transformed yeasts were diluted and grown in SD/-LTH control medium and in SD/-LTHU selective medium for 3 days at 30 °C. (**D**) Volcano plots showing the distribution of all quantified proteins after filtering in Arabidopsis GFP immunoprecipitation experiments. Samples are represented after conducting a Student’s *t*-test based on label-free quantification (LFQ) values, with their corresponding protein abundance ratios (GFP/RIRG190). The cutoff curve indicates proteins that are significantly more associated with free GFP (left) and RIRG190 (right) in accordance with their FDR (FDR = 0.01/0.05, S0 = 0.1). (**E**) Primary root length of Arabidopsis Col-*0* WT and *thal−/+* heterozygous mutants. Root systems were measured 14 days after sowing. Values are means of four biological repeats (n = 55–69; ****, *p* < 0.0001; One-way ANOVA followed by multiple comparison (α < 0.05)). (**F**) Meristem cell number of Arabidopsis Col-*0* WT and *thal−/+* mutant roots six days after sowing. The number of cortical cells between the quiescent center and the first elongated cell was counted. To increase the accuracy of the data, we individually counted the right and left side rows of cortical cells in the root meristem to obtain an average value per analyzed root. Data are means ± SEM of 19 average values (****, *p* < 0.0001; One-way ANOVA followed by multiple comparison (α < 0.05)). (**G**) Confocal laser-scanning microscopy pictures of Arabidopsis root tips incubated with 1% propidium iodide six days after sowing. Bar, 50 µM. Green arrowheads indicate the end of the quiescent center, and white arrowheads mark the first elongated cell.

Because of the observed effect of *RIRG190* expression in Arabidopsis, we tested whether the RIRG190–SAS10 nuclear association is conserved in Arabidopsis. In Arabidopsis, SAS10 plays a role in ribosome biogenesis by influencing rRNA expression and processing through its association with the SSU processome core component M-phase phosphoprotein 10 (MPP10), and with the histone chaperone nucleolin 1 (NUC1) [68,70,73]. Hence, the binary interaction of the AtSAS10 with RIRG190 was examined by means of Y2H. AtSAS10 did not show autoactivation, and a positive interaction was observed between RIRG190 and AtSAS10 (Figure 6A). Next, in tomato, the data confirmed the interaction between SlSAS10 and its SlMPP10 homolog, but RIRG190 did not directly interact with MPP10 (Figure 6B). To determine whether RIRG190 and MPP10 might be part of the same protein complex through the association with SAS10, a yeast three-hybrid (Y3H) assay was performed. Strong protein interaction between RIRG190 and SlMPP10 was only detected in the presence of SlSAS10, and this trimeric interaction was also weakly observed for Arabidopsis (Figure 6C). Hence, RIRG190 might interact with the SAS10/MPP10 complex in tomato and to some extent in Arabidopsis.

As we unraveled the ability of RIRG190 and SAS10 to form a protein complex in yeast with a known SAS10 interactor, we investigated whether RIRG190 can form similar protein complexes in planta. To test this, we conducted a GFP immunoprecipitation in 14-day-old roots of *RIRG190-GFP* Arabidopsis homozygous lines (Figure 6D). As a control for off-target identification, Arabidopsis *GFP* roots were used. MaxQuant protein files from transgenic roots were analyzed in the Perseus software, and the Pearson correlation quality control ranged from 0.85 to 0.96, indicating good reproducibility among samples (Figure S8A). A total of 19 Arabidopsis proteins were significantly more enriched in *RIRG190-GFP* roots compared to *GFP* (FDR 0.05/S0 = 0.1) (Dataset S1B). Out of these 19 Arabidopsis candidates, more than half of the proteins were related to translation (cfr. ribosomal proteins), and, interestingly, the known AtSAS10 nuclear interactor AtNUC1, which participates in chromatin regulation of rDNA variants located in nucleolar organizer regions [70], was found. Although the GO analysis of RIRG190-associated proteins did not show any significant enrichment, Cytoscape visualization of the RIRG190-interacting network pointed out the clustering of proteins taking part in RNA splicing, rRNA processing, translation, DNA-dependent transcription regulation, vesicle trafficking, and nuclear transport (Figure S8B).

Altogether, these results demonstrate that RIRG190 might be taking part in similar processes as the ones described for SAS10 proteins, such as rRNA processing, ribosome biogenesis, and chromatin remodeling.

### 2.7. RIRG190 and SlSAS10 Might Control Cell Division and Endoreduplication

*AtSAS10* mutants have been previously reported to exhibit abnormalities in embryo cell patterning due to an unbalanced rRNA processing and expression [70]. Available single-cell transcriptomic data in Arabidopsis roots further indicate a main expression of *AtSAS10/THAL* in initial stem cells as well as in dividing cells of Arabidopsis roots, suggesting a putative role in rRNA biogenesis to facilitate cell cycle-related processes [74]. Because cell cycle and rRNA biogenesis are conserved events required to support cell activity, we studied the role of AtSAS10 in cortical cell division by studying the root growth of two independent *atsas10*/*thal* heterozygous mutant lines (*thal-1/+*, SALK_016916.20.75; *thal-2/+*, SALK_036872.54.10), as *atsas10*/*thal* homozygous mutants are embryo lethal [70]. After confirming the significant downregulation of *AtSAS10/THAL* in both *thal−/+* mutant lines by RT-qPCR analysis (Figure S9), we quantified the root length of 14-day-old *thal−/+* mutants. Average root lenghts were 5.28 ± 1.10 in Col-0, 6.49 ± 0.85 in *thal-1/+*, and 6.28 ± 0.90 in *thal-2/+*. Compared to Col-0, *thal-1/+* exhibited a mean increase of 1.21 (1.23-fold), while *thal-2/+* showed a mean increase of 1.00 (1.19-fold) (Figure 6E). Meristematic cortical cell analysis (Figure 6G) revealed that the partial absence of *AtSAS10* phenocopied the overexpression of *RIRG190* in roots (Figure 3D,E), also displaying a significantly increased number of meristematic cortical cells in the root apical meristem (Figure 6F). Average cortical cell number was 30.7 ± 2.0 in Col-0, 38.3 ± 2.5 in *thal1/+*, and 36.3 ± 1.8 in *thal2/+*. Compared to Col-0, *thal1/+* exhibited a mean increase of 7.63 (1.25-fold), while *thal2/+* showed a mean increase of 5.61 (1.18-fold).

Mycorrhizal roots display a mixed population of cortical cells during AM symbiosis [7]. These include smaller ‘split cells’ formed prior to arbuscule accommodation by activation of mitosis, as well as larger arbusculated and neighboring cells undergoing endoreduplication [7,15]. Based on the collected data described above, we hypothesized that RIRG190 might influence SAS10 activity to boost the metabolism, e.g., to activate the cortical cells to promote fungal infection. Thus, we conducted a ploidy analysis in 4-week-old non-mycorrhized tomato *RIRG190-GFP*, *GFP-SAS10*, and *GFP* roots and investigated the endoreplication index. Indeed, the tomato roots overexpressing *RIRG190-GFP* and *GFP-SlSAS10* fluorescent fusions displayed a significantly higher proportion of polyploid cells compared to *GFP* control roots (Figure S10).

These data could indicate that AtSAS10 might be involved in the establishment of a set of metabolically active cortical cells that enable colonization.

## 3. Discussion

Here, we combined protein interactomic, transcriptomic, and reverse genetic approaches to shed light onto the molecular mechanisms by which the secreted nuclear-localized effector RIRG190 impacts AM symbiosis (Figure S11). AMF genomes have been described to contain hundreds of genes that encode effector proteins, which are expected to play an important role in the communication between the macro- and microsymbiont during AM symbiosis [32,34,75,76]. This symbiosis is maintained through the sustained formation of differentiated fungal structures hosted inside root cortical cells, the arbuscules, in which nutrients, but potentially also effector proteins, are delivered [41,77]. RIRG190 has been annotated as a putative SCP-like extracellular protein of the PR1-like family and is highly expressed in the extraradical mycelium of *R. irregularis* during its interaction with *M. truncatula*, but lower transcript levels have also been observed in arbusculated root cells [34]. In this study, the data demonstrate that RIRG190 can be secreted based on its SP and predominantly localizes in the nucleus when expressed in planta, while most SCP/CAP domain proteins have been characterized as functioning in the plant apoplast [58,59,60]. This observation is rather surprising and further investigation using a secreted variant of the RIR190 effector, coupled with functional analysis of its role during symbiosis, could help determine whether it also serves an extracellular function.

Nevertheless, we found a clear nuclear localization inside the nucleus of both Arabidopsis roots, tomato roots, and tobacco leaves, and strong interaction with nuclear proteins in both the Y2H screen and GFP immunoprecipitation, strongly suggesting a role for RIRG190 in the plant nucleus. NLS deletion variants of RIRG190 can aid in determining whether RIRG190 translocation to the nucleus is canonically mediated by the α-importin system and results in the observed phenotypes, or if, in contrast, the small molecular weight of the effector facilitates passive diffusion through the nuclear pore. In addition, the expression of *RIRG190-GFP* in the rice hemibiotrophic fungus *Magnaporthe oryzae* or the tomato filamentous fungus *Fusarium solani* strain K could serve as an alternative approach to validate the in vivo functional secretion, translocation, and nuclear compartmentalization of the RIRG190 effector [37].

The precise timing of the secretion of this effector during the establishment of the symbiosis remains elusive and is difficult to assess due to the absence of valid approaches to genetically modify AMF [36]. Yet, the implementation of immunohistochemistry, proteomics, and spatial or single-cell RNA-Sequencing in mycorrhized tomato roots could uncover the precise spatial and temporal localization of the endogenous RIRG190 effector protein or transcript, respectively. Nevertheless, detailed transcriptional analysis of *SlPT4p:GFP* root material confirmed its expression in arbusculated and in arbuscule-deprived tomato root segments, indicating that the effector might be secreted from symbiotic and pre-symbiotic fungal structures.

We identified the nuclear protein SlSAS10 as an RIRG190 interactor. SAS10 proteins are conserved eukaryotic proteins that have been described to participate in rRNA processing, ribosomal biogenesis, rDNA chromatin condensation, nucleolar architecture, and gene silencing among different kingdoms [68,69,70]. rRNA processing is an event occurring within the nucleolus of cells. In agreement, SlSAS10 as well as RIRG190 were detected inside nuclear substructures resembling nucleoli when expressed in *N. benthamiana* leaves and tomato roots.

A role for SAS10 proteins in ribosome biogenesis by influencing rRNA expression and processing was proposed through their association with MPP10 and NUC1 [68,70,73]. SAS10 has also been shown to condition the nucleolar localization of MPP10 and other associated proteins in zebrafish, indicating its ability to mobilize other proteins into the nucleus [68]. We could confirm that the tomato and Arabidopsis SAS10 homologs could interact with MMP10, further providing evidence that the function of SAS10 is conserved in the animal and plant kingdoms. Additionally, the Y3H assay demonstrated that the effector RIRG190 can form a complex with MMP10 through SAS10 in the investigated plant species. What is more, NUC1, the known AtSAS10 interactor known to participate in pre-rRNA processing and nucleolar organization, was identified among the enriched proteins in the Arabidopsis RIRG190-GFP immunoprecipitated fraction [78,79]. Altogether, these data provide strong evidence that *Rhizophagus* secretes RIRG190 to impact rRNA biogenesis through binding with the SAS10–MMP10 complex. Overexpression of *RIRG190* or partial silencing of *AtSAS10* led to an increased meristematic cortical cell number in Arabidopsis roots. These changes in cell patterning have also been observed in Arabidopsis *thal−/+* embryos, suggesting that AtSAS10 might be negatively influencing cell division [70]. However, additional experiments, such as coimmunoprecipitation, are necessary to further confirm the interaction between RIRG190 and AtSAS10 and to validate the role of the RIRG190–SAS10–MPP10 complex in regulating ribosome biogenesis during the AM symbiosis.

Although alteration of the cell cycle is widely observed in plant hosts engaging in microbe interactions, the detailed molecular mechanisms and functional implications of this activity remain to be identified [80,81,82,83,84]. Some cells can re-replicate their DNA without the subsequent cell division, resulting in increased ploidy levels to boost the metabolism [85,86,87]. Both ectopic cell cycle activation coupled with cytokinesis prior to arbuscule accommodation, as well as somatic polyploidization of cortical cells in mycorrhized tissues, have been described [7,15,16]. In this work, the data shows that both tomato roots overexpressing *SlSAS10* and those that ectopically express *RIRG190* have higher endoreplication levels. Additionally, mutation in *AtSAS10* as well as *RIR190* overexpression, stimulated root cell divisions in Arabidopsis. Interestingly, *RIRG190* overexpression resulted in higher m% and a%, while overall colonization frequency (F%), intensity (M%), and global arbuscule abundance (A%) remained unaffected, suggesting that colonized cortical fragments were more densely filled with fungal structures and contained a greater proportion of arbuscules; consistent with this, *PT4* expression was elevated, indicating that these denser colonization events are functionally active and that RIRG190 enhances the local intensity of AM symbiosis without altering its overall frequency. On the contrary, roots in which *SlSAS10* levels were decreased or increased, displayed severe defects in arbuscule establishment and fungal colonization, underscoring the requirement for finely tuned SAS10 activity in AM symbiosis.

In Arabidopsis, *AtSAS10* gene expression is high in dividing and in initial root cell lineages that act as ‘stem cell’ reservoirs to maintain the root apical meristem architecture [74]. Additionally, tomato *SlSAS10* promoter activity in the absence of the fungus was detected in highly dividing cell tissues with a redirection to arbusculated cells during mycorrhization, which are hallmarked by endoreduplication traits [15]. Therefore, it is tempting to speculate that a specific spatio-temporal *SlSAS10* expression is necessary in cortical cells to confer dividing capacities to differentiated cortical cells during AM symbiosis. We therefore propose that SAS10, through its role in ribosome biogenesis, maintains the translational capacity required for cortical cell proliferation. By modulating SAS10 activity, RIRG190 may ensure sufficient protein synthesis to sustain the cellular remodeling associated with AMF colonization in tomato, while in Arabidopsis the same increase in ribosome biogenesis supports elevated cell division, resulting in longer roots even in the absence of symbiosis. This suggests a conserved mechanism in which RIRG190–SAS10 interaction couples ribosome biogenesis to both root architectural changes and symbiotic cortical cell differentiation. In the future, the generation of stable tomato lines in which *SlSAS10* is knocked out by CRISPR editing or *RIRG190-GFP* or *SlSAS10-GFP* are ectopically expressed, could validate the observed phenotypes.

Our findings provide additional evidence that, beyond immunity-related mechanisms exemplified by effectors such as SP7, which modulates host immunity through interaction with ERF19 or RiNLE1, which targets histone H2B to suppress defense responses, RIR190 acts through a distinct mechanism. RIRG190 complements the known effector repertoire by linking effector activity to fundamental cellular processes that sustain the root cortical cell physiology for arbuscule accommodation, thereby diverging from immunity-focused effectors and broadening our understanding of how AMF effectors can reprogram host physiology to orchestrate the symbiosis. Endoreduplication is often observed in metabolically active cells requiring high levels of ribosomes for protein synthesis. During the establishment of AM, cortical cells need to be reactivated to accommodate the fungus. Additionally, arbuscule-containing cells are the engines of the symbiosis, necessitating massive protein activity to control the nutrient exchange and fungal accommodation. How RIRG190 exactly impacts SAS10 function, ultimately leading to possible changes in rRNA biogenesis and changes in the cell cycle, is an intriguing subject to be tackled in the future.

## 4. Materials and Methods

### 4.1. Plant and Fungal Material and Growth Conditions

#### 4.1.1. Tomato

For the generation of composite plants, tomato cv MoneyMaker seeds were surface-sterilized by soaking in 2.35% *w*/*v* sodium hypochlorite for 10 min, followed by three consecutive washes with sterile water. Tomato sterile seeds were placed in Petri dishes containing wet sterile cotton disks and grown at 24 °C in dark conditions for three days, after which they were transformed with the desired plasmids via *Agrobacterium rhizogenes* [88,89]. After 4 weeks, composite plants were transferred to 1.5 L round pots containing autoclaved sterilized sand:vermiculite mixture (1:1 *v*/*v*) with or without approximately 250 spores of *Rhizophagus* (SYMPLANTA GmbH & Co. KG, Darmstadt, Germany). Tomato plants were grown at 24 °C under long-day conditions (16 h/8 h photoperiod). To promote mycorrhization, inoculated and non-inoculated plants were supplied every 3.5 days with 30 mL of Hewitt solution containing 25% (325 µM) of the standard phosphorus concentration [90].

#### 4.1.2. Arabidopsis

Homozygous seeds of Arabidopsis *RIRG190-GFP* (*RIRG190-GFP.1* and *RIRG190-GFP.2*) stable overexpression lines were produced in this work following the floral dip method [91]. Heterozygous *atsas10*/*thal*, *thal-1/+* (SALK_016916.20.75) and *thal-2/+* (SALK_036872.54.10) mutants were obtained from the Nottingham Arabidopsis Stock Centre (NASC). The two single-loci Arabidopsis *GFP-THAL* overexpression lines were kindly donated by Dr. Guang-Yuh Jauh (Institute of Plant and Microbial Biology, Academia Sinica, Taipei, Taiwan) [70]. Arabidopsis seeds were surface-sterilized using chlorine gas and stratified for 48 h at 4 °C in dark conditions. Seeds were sown and vertically grown on ½ MS agar plates (2.15 g/L MS, 0.5 g/L MES, 10 g/L sucrose, 0.1 g/L myo-Inositol, 10 g/L plant tissue culture agar, 1 L of distilled water; pH 5.7) at 21 °C under 16 h/8 h light conditions. Three-week-old tobacco plants were grown at 23 °C and 60% relative humidity in the greenhouse with a 10 h/14 h photoperiod.

### 4.2. Bacterial Strains and Culture Conditions

For plasmid production, *Escherichia coli* DH5α strain were transformed using heat shock and cultured in Luria–Bertani Broth (LB) medium (10 g/L tryptone, 5 g/L of yeast extract, 10 g/L of sodium chloride, 1 L of distilled water; pH 7.0) supplemented with the corresponding antibiotics. Plasmids were introduced through electroporation in *Agrobacterium tumefaciens* C58C1, *A. rhizogenes* ArquaI or *A. rhizogenes* ATCC15834. *Agrobacterium* strains were kept as glycerol stock and grown freshly before use at 28 °C on plates with Yeast Extract Beef (YEB) agar medium (5 g/L Beef extract, 5 g/L peptone, 5 g/L sucrose, 1 g/L yeast extract, 0.3 g/L magnesium sulfate, 10 g/L select agar, 1 L of distilled water; pH 5.7) or in liquid YEB cultures lacking agar supplemented with the corresponding antibiotic. To create a bacterial culture, strains were grown overnight in liquid YEB medium containing the specific antibiotic at 28 °C under shaking conditions. Cultures were centrifuged at 2500 rcf for 10 min and the bacterial pellets were resuspended in infiltration buffer (9.76 g/L MES, 4.76 g/L magnesium chloride, 0.98 g/L acetosyringone; pH 5.6) and diluted to an optical density (OD_600_) of 1.

### 4.3. Fungal Strain

Plants were treated with approximately 250 spores of *Rhizophagus irregularis* DAOM197198 (SYMPLANTA GmbH & Co. KG, Darmstadt, Germany).

### 4.4. Plasmids

For monitoring the transformation of tomato composite plants, the previously published fluorescent screening module was used [42,92].

#### 4.4.1. Golden Gate Expression Vectors

Constructs were produced via Golden Gate cloning technology [92,93,94]. The *SlSAS10* (Solyc11g072390.1) CDS, the *RIRG190* (POG59785.1; U9THM5) CDS lacking the predicted SP, and the 1638 nucleotides upstream the START codon of the *SlPT4* gene (Solyc06g051850.2) corresponding to the *SlPT4* promoter region were PCR-amplified using Q5 High-Fidelity DNA Polymerase (New England Biolabs, Ipswich, MA, USA) from mycorrhized tomato root cDNA for CDSs or genomic DNA for promoter isolation. Green Gate level 1 modules containing the above-mentioned sequences were generated via Gibson assembly, verified by Sanger sequencing, and further assembled via Golden Gate to generate the following constructs: *35Sp:RIRG190-GFP*, *35Sp:GFP-SlSAS10*, *35Sp:GFP*, *SlPT4p:GFP*, *pFAST 35Sp:RIRG190-GFP*, and *pFAST 35Sp:GFP* [92,93,94].

#### 4.4.2. Reporter Lines

For the study of *SlSAS10* transcriptional activity, the 3 kb upstream region corresponding to the putative *SlSAS10* promoter was PCR-amplified from tomato genomic DNA and combined into the PGGA Green Gate entry 0 module. The resulting module and the modules containing the GUS CDS, the 35S terminator, and a terminal linker were assembled into the Golden Gate destination vector PGGPAG to produce the *SlSAS10p:GUS* and 35Sp:*GUS* vectors.

#### 4.4.3. Gateway Expression Vectors

For the generation of constructs used for subcellular localization studies in tobacco, the Gateway cloning technology (Invitrogen, Waltham, MA, USA) was preferred. Gene CDSs were PCR-amplified from plant cDNA using specific primers, recombined into pDONR207, pDONR221 or pDONR221 2in1 and verified by Sanger sequencing. The CDSs were further ligated into Gateway binary vectors using the LR clonase (Invitrogen, USA). For the generation of the *35Sp*:*SlSAS10* RNA interference (RNAi) construct, the *SlSAS10* cDNA region between nucleotide 233 and 384 was PCR-amplified, and the purified 151 bp DNA fragment was further ligated into the pDONR207 vector and subsequently recombined into the pK7GWIWG2(II)-RedRoot destination vector via the Gateway technology [95]. As a negative control, the empty pK7GWIWG2(II)- RedRoot vector (RNAi EV) was used.

All cloning primers and gene accessions are listed in Table S1A, and the specific composition of all generated vectors can be found in Table S1B.

### 4.5. RIRG190 In Silico Analysis and Protein Homology-Based Tree Generation

The presence of an N-terminal SP in the RIRG190 effector protein sequence was investigated by the SignalP 6.0 online tool (https://services.healthtech.dtu.dk/service.php?SignalP, (accessed on 22 May 2024)), the in silico effector was predicted using EffectorP 3.0 (https://effectorp.csiro.au/, (accessed on 22 May 2024)), and the presence of an NLS was determined via LOCALIZER (https://localizer.csiro.au/, (accessed on 22 May 2024)).

To build a protein homology-based tree, the RIRG190 FL protein sequence was blasted against the non-redundant protein database in NCBI. Subsequent homologous effector-like protein sequences were subjected to the above-mentioned selection criteria and selected as described previously [42]. The resulting ten homologous candidate effector amino acid sequences were aligned and a pairwise sequence comparison was performed. To infer the phylogenetic relationship among the different nuclear-localized effector-like candidates, the neighbor-joining was applied as a distance-based reconstruction method and clade robustness was achieved using 1000 bootstrap replicates using CLC Workbench 8.1 software (Qiagen, Aarhus, Germany) [96]. To identify homologous protein domains among the different SAS10 proteins investigated in this work, a pairwise sequence comparison was also performed. Results from all the RIRG190 effector in silico predictions can be found in Table S1C.

### 4.6. Total RNA Isolation

Total mRNA was extracted from ground root tissue using the ReliaPrep™ RNA Miniprep System according to manufacturer’s instructions (Madison, WI, USA), and the RNA concentrations were measured with an ND-1000 spectrophotometer (Thermo Fisher Scientific Nanodrop, Waltham, MA, USA). For single-stranded cDNA synthesis, 1 µg of total RNA was reverse-transcribed using the iScript cDNA synthesis kit as described by the manufacturer (Bio-Rad Laboratories N.V., Hercules, CA, USA). Oligonucleotides were retrieved from literature or designed with the Primer3plus online tool using the C-terminal region of the gene sequences as input (https://www.bioinformatics.nl/cgi-bin/primer3plus/primer3plus.cgi, (accessed on 22 May 2024)) and target specificity was investigated by Primer-blast tool (https://www.ncbi.nlm.nih.gov/tools/primer-blast/, (accessed on 22 May 2024)).

### 4.7. Real-Time Quantitative Reverse Transcription PCR (RT-qPCR) Analysis

RT-qPCR reactions for a given gene and template were conducted in triplicates on 384-well plates. For each reaction mixture, a total of 5 µL sample was investigated, containing the Fast SYBR Green Master Mix (Applied Biosystems, Illkirch, France), 10% input cDNA, and a final concentration of 0.25 μM for each primer. RT-qPCR reactions were run using the Roche Lightcycler 480 system (Roche Diagnostics, Diegem, Belgium) as follows: 1× preincubation (95 °C for 5 min), 45× amplification (95 °C for 10 s, 60 °C for 10 s and 72 °C for 10 s), 1× melting curve (95 °C for 5 s and 65 °C to 97 °C for 1 min), and 1× cooling down (40 °C for 10 s). Transcript levels were normalized using *SlEF1α* (Solyc06g009960.1) and *SlGAPDH* (Solyc05g014470.2) housekeeping genes for tomato, *AtACTIN* 2 (At3g18780) and *AtTUBULIN* 2 (At5g62690) for Arabidopsis, and *RiEF1α* (ABB90955.1) for *RIRG190* [19,97]. Relative fold changes were calculated according to the delta-delta Ct method (2^ΔΔCt^) after normalization using the respective housekeeping genes. All RT-qPCR primers used in this study can be found in Table S1D.

### 4.8. Yeast Secretion Trap (YST) Assay

The nucleotide sequences encoding the putative RIRG190 effector SP, the CDS lacking the SP (CDS-SP), and the FL effector sequence were PCR-amplified using EcoRI-NotI restriction sites. The digested cDNA fragments were further ligated into the pYST1 vector to produce an in-frame fusion with the *SUCROSE INVERTASE 2* (*SUC2*) gene lacking its endogenous SP [57]. Yeast reporter strain Y02321 (Euroscarf, Scientific Research and Development GmbH, Oberursel, Germany) was transformed using the standard lithium acetate/single-stranded carrier DNA/polyethylene glycol method. Yeast transformed with the empty pYST1 vector (EV) was used as a negative control, while the Medicago CLAVATA3/ESR (CLE)-related protein 13 (*MtCLE13*) gene fused to *SUC2* was used as a positive control [88,98,99]. Positively transformed yeast colonies were selected on SD/L plates (26.7 g/L synthetic-defined medium, 0.69 g/L leucine drop-out (Clontech, Saint-Germain-en-Laye, France), 2% select agar, 1 L distilled water), and DNA insertion verification was performed by PCR amplification. Serial dilutions were dropped on control SD/L agar medium and on sucrose selective agar medium YNB/LS (6.7 g/L yeast N base without amino acids, 0.69 g/L drop-out minus leucine (Clontech, France), 2% sucrose, 2% select agar, 1 L distilled water). Yeast-containing agar plates were incubated upside down at 30 °C for 3 days, after which protein secretion was assessed. Primers used for the YST cloning are listed in Table S1A.

### 4.9. Confocal Microscopy

The constructs *35Sp:RIRG190-GFP*, *35Sp:CFP*-*SlUNK* and *35Sp:CFP*-*SlSAS10* were transiently expressed in tobacco leaf epidermal cells by *A. tumefaciens* (C58C1 strain)-mediated transformation [100] and fluorescent emission was visualized with a Zeiss LSM 710 inverted confocal microscope (Oberkochen, Germany) under an excitation laser of 488 nm or 405 nm for GFP or CFP signal detection, respectively. For subcellular colocalization studies, *35Sp:CFP*-*SlUNK* and *35Sp:CFP*-*SlSAS10* were coinfiltrated with the *35Sp:RIRG190-GFP* construct as described [100]. Plant material was imaged in sequential mode at 48 h post-infiltration using the above-mentioned tools. For the detection of GFP fluorescence in tomato composite plants or in Arabidopsis homozygous stable lines, roots were mounted on slides with distilled water and examined under the same confocal microscope using the 488 nm excitation laser.

### 4.10. Ectopic Gene Expression in Tomato Composite Plants

To produce composite plants, sterilized tomato seeds were transformed as described above. Sectioned roots were infected by coating the freshly cut surface with an *A. rhizogenes* ArquaI strain agar culture carrying *35Sp:RIRG190-GFP* or *35Sp:GFP.* Additionally, tomato composite plants carrying the *35Sp:GFP-SlSAS10*, the *SlSAS10p:GUS*, the *SlSAS10* RNAi, and the RNAi EV were generated. For the creation of *SlSAS10* RNAi roots, a minimum of 200 tomato seedlings were transformed to guarantee enough silenced composite plants per biological repeat. All transformed plantlets were screened weekly under the fluorescence microscope for constitutive red fluorescent signal emitted by the *mRuby:NLS* or red fluorescent protein (RFP) present in the RNAi vector and WT roots were removed. A minimum of six transformed composite plants for each plant species and biological repeat were transferred to pots at 28 days post-transformation. Plants were grown as described above and root material was gathered at the desired time points.

### 4.11. Estimation of Rhizophagus Root Colonization

To visualize mycorrhizal structures, plant host root systems were stained using ink as described by Vierheilig et al. [101]. Estimation of the intensity of the root cortex colonization was carried out according to the Trouvelot method [61]. The mycorrhization frequency (F%) and intensity (M%) and the arbuscule abundance (A%) in the whole root system, and the mycorrhization intensity (m%) and the arbuscular abundance (a%) in mycorrhized root fragments were measured using the Mycocalc software (https://www2.dijon.inrae.fr/mychintec/Mycocalc-prg/download.html, (accessed on 22 May 2024)). For each biological repeat, a minimum of 30 root pieces per biological repeat and construct were analyzed under the Leica stereo microscope (Wetzlar, Germany).

### 4.12. Wheat Germ Agglutinin (WGA) Fluorescent Staining

Root systems from composite plants expressing the *35Sp:GFP*, *35Sp:RIRG190-GFP, 35Sp:GFP-SlSAS10*, and *SlSAS10p:GUS* fusion proteins were subjected to WGA staining using 10 µg/mL of WGA-Alexafluor 488 conjugated dye (Thermo Fisher Scientific) diluted in PBS [102]. Briefly, root systems were sampled and incubated in 50% ethanol for 7 days at room temperature, after which they were incubated for 45 min at 90 °C in 10% KOH, followed by three consecutive washes of five min each with distilled water. Roots were incubated for 2 h at room temperature in 0.1 M HCl solution and consequently rinsed three times with PBS. Resulting root systems were incubated for a minimum of 2 h in WGA solution at 4 °C in a dark environment. Samples were mounted on slides with 50% glycerol and subjected to confocal scanning microscopy. At least nine transformed roots from independent plants were studied under the confocal laser-scanning microscope Zeiss LSM 710. Z-stack images were acquired in sequential mode, using 488 nm excitation and an emission window between 488 and 511 nm for GFP detection. Detailed images were created using the 3D tool of the ZEN 3.5 blue edition software.

### 4.13. Construction of Arabidopsis Lines

To generate stable homozygous Arabidopsis lines carrying the effector fusion, plants were transformed as previously reported [91]. Transgenic seeds carrying the *pFAST 35Sp:RIRG190-GFP* or *pFAST 35Sp:GFP* insertion were selected based on the fluorescence-accumulating seed technology system as described previously [103]. Single-loci insertions were selected at the T2 population, and experiments were performed with the homozygous T3 generation. Root genomic DNA was extracted using the DNeasy plant mini kit (Qiagen, Hilden, Germany) and the genomic insertion was PCR-validated. Primers used for the T-DNA insert validation are listed in Table S1E.

### 4.14. Arabidopsis Phenotypic Analysis

Primary root length was analyzed from the root systems of 14-day-old seedlings grown vertically at 21 °C under long-day conditions (16 h/8 h photoperiod). Root systems were photographed, and pictures were analyzed using the NeuronJ plugin in ImageJ software (http://rsb.info.nih.gov//ij/, accessed on 22 April 2024) to determine the root length [104].

To quantify the meristematic cortical cells, roots from six-day-old Arabidopsis seedlings were stained with 1% propidium iodide, mounted on slides in distilled water and studied under a confocal laser-scanning microscopy Zeiss LSM 710. Images were acquired in sequential mode, using 561 nm excitation and an emission window between 519 and 643 nm for propidium iodide detection. The number of cortical cells between the quiescent center and the first elongated cell was counted. To increase the accuracy of the data, we counted individually the right and left side rows of cortical cells in the root meristem to obtain an average value per analyzed root [62].

### 4.15. Y2H cDNA Library Screening

The Y2H cDNA library screening assay was performed as previously described [105]. The pDONR221 containing the CDS of *RIRG190* lacking the endogenous SP was recombined into the PGBKT7 bait vector via the Gateway technology (Invitrogen, USA). To exclude bait autoactivation, the PGBKT7 RIRG190 was cotransformed with the empty PGADT7 prey vector in the reporter yeast strain PJ69-4α [105]. The Y2H library screening using the PGBKT7 RIRG190 competent yeasts was conducted as previously reported [42].

### 4.16. Y2H Pairwise and Y3H Assays

The pDONR207 or pDONR221 containing the gene of interest was recombined into the prey vector PGADT7 and/or bait vector PGBKT7 following the Gateway cloning standard procedures (Invitrogen, USA). To evaluate binary interaction, bait and prey were cotransformed and grown in selective medium as described [96]. For the Y3H assay, the pEN-R2-NLS-3xMyc-L3, the pDONR207 carrying *SlSAS10* or *AtSAS10*, and the pEN-L4-pGPD-R1 were recombined into the destination vector pMG426 via Multisite Gateway LR reaction (Invitrogen, USA). Yeast cotransformation was performed using the PGBKT7 *RIRG190*, PGADT7 *SlMPP10/AtMPP10*, and the PMG426 *AlSAS10/AtSAS10* vectors. As a negative control for the interaction, the empty PMG426 vector was used for the cotransformation. Yeast serial dilutions were dropped on SD/-LTH control medium, while interactions were assessed in SD/-LTHU selective medium (26.7 g/L synthetic-defined medium, 0.6 g/L drop-out mix of leucine, tryptophan, histidine and uracil [Clontech, France], 2% select agar).

### 4.17. rBiFC Assay

#### 4.17.1. rBIFC Construct Generation

To investigate the interaction between RIRG190 and the tomato protein of unknown function (SlUNK, Solyc07g045450) and the homologous SAS10 proteins (SlSAS10, Solyc11g072390, and its isoform Solyc11g072320), the respective gene CDSs were cloned into pDONR221 2in1 entry vectors. The 2-in-1 N-terminal rBiFC expression clones were generated by combining the *SlUNK* and the *SAS10* homologous genes with *RIRG190* [71]. As a positive control, the rBiFC expression clone containing the Arabidopsis *AtSKP1* (*At1g75950*) and *AtMAX2* (*At2g42620*) gene pair was used [72]. As a negative controls, the known interactor of the nuclear effector GLOIN707, Sl296, was tested for the interaction with RIRG190, whereas SlSAS10 was co-expressed with GLOIN707 [42]. All studied genes were fused in the same N-terminal position to avoid tag interference.

#### 4.17.2. rBIFC Confocal Analysis

Subcellular localization of the in vivo interaction was studied by *Agrobacterium tumefaciens* (C58C1 strain)-mediated transformation in tobacco leaves, as previously described [100]. rBiFC images were obtained using a Zeiss LSM 710 confocal microscope employing the white-light laser with a 40×/1.2 water-immersion objective. Images were acquired in sequential mode, using 513 nm excitation and an emission window between 519 and 550 nm for YFP detection, and 555 nm excitation and an emission window between 578 and 620 nm for RFP detection. All images were acquired under the same settings. The plant cell nucleus was delimited by the round contour tool of the ZEN 3.5 blue edition software, after which the average intensity of the RFP and YFP channels was selected. All images were devoid of saturated pixels [106].

### 4.18. Spatial Analysis of SlSAS10 Transcriptional Activity

To localize the endogenous expression of *SlSAS10*, the transcriptional fusion of the *SlSAS10* promoter with the *β-glucuronidase* (*GUS*) reporter gene, as well as the control vector *35Sp:GUS*, was generated, and the promoter-*GUS* activity was studied in mycorrhized and non-mycorrhized tomato composite plants at four wpi. To measure the *GUS* activity, root systems were harvested in NT buffer (12.12 g/L tris(hydroxymethyl)aminomethane [Tris], 2.92 g/L Sodium chloride [NaCl], 1 L of distilled water), transferred to GUS buffer (1.044 g/L X-gluc dissolved in dimethyl sulfoxide [DMSO], 0.64 g/L potassium ferricyanide [K_3_[Fe(CN)_6_)] in NT buffer) and incubated overnight at 37 °C in dark conditions. Subsequently, roots were subjected to WGA costaining using 10 µg/mL of WGA-Alexafluor 488 conjugated dye (Thermo Fisher Scientific) as described above. For *GUS* detection, root samples were mounted on slides in 50% glycerol and visualized by light microscopy using the Olympus BX51 microscope (Tokyo, Japan) or the Leica stereo microscope, and subsequent pictures were obtained.

### 4.19. GFP Immunoprecipitation and Liquid Chromatography Tandem-Mass Spectrometry (LC MS/MS) and Gene Ontology Analysis

To identify RIRG190 protein interactors in Arabidopsis, a GFP pull-down was performed in 2-week-old stably transformed Arabidopsis roots ectopically expressing *35Sp:RIRG190*-*GFP* or *35Sp:GFP*. GFP immunoprecipitation and sample preparation were performed as previously described [107] using 2 g of crushed root material per biological replicate. Peptides were detected with the Q Exactive HF Mass Spectrometer [107].

Raw peptide data of *35Sp:GFP* and *35Sp:RIRG190-GFP* files were analyzed with MaxQuant from the Galaxy online platform using standard parameters found in Table S1F. Proteins identified with at least one unique peptide were retained. The false discovery rate (FDR) for peptide and protein identifications was set to 1, and the minimum peptide length was set to seven amino acids. MaxQuant protein group files were loaded in Perseus (version 1.6.15) and proteins identified by contaminant, reverse, and site were removed. Samples were grouped by the respective triplicates and filtered for a minimum of three valid values per triplicate to strengthen the search. Missing label-free quantification (LFQ) values were imputed from a normal distribution using standard settings in Perseus. A Student’s *t*-test analysis was performed, and a volcano plot was built using permutation-based FDR to determine the significantly enriched proteins in the *35Sp:RIRG190-GFP* samples compared to those identified in the *35Sp:GFP* roots. Two cutoffs were applied for significance, FDR = 0.05/S0 = 0.1 and FDR = 0.01/S0 = 0.1. STRING protein–protein interaction files from each of the significantly enriched proteins found in the Arabidopsis *RIRG190-GFP* samples were downloaded, and a RIRG190 protein network visualization was built using Cytoscape 3.9.1 software [108,109,110].

### 4.20. Plant Protein Extraction and Immunoblot Analysis

For effector fusion protein detection in different plant material, i.e., tobacco, tomato and Arabidopsis, 200 mg of crushed leave/root material from each line were subjected to protein extraction buffer (23.63 g/L Tris (THAM) hydrochloride [Tris-HCl] pH 7.5, 8.76 g/L NaCl, 10% glycerol, 2.9 g/L Ethylenediaminetetraacetic acid [EDTA], 0.21 g/L sodium molybdate [Na_2_MoO], 0.042 g/L sodium fluoride [NaF], 1.54 g/L dithiothreitol [DTT], 1% (*v*/*v*) NP-40, 0.5% (*v*/*v*) polyvinylpolypyrrolidone [PVPP], protease inhibitor cocktail [Roche, Basel, Switzerland], 1 L of distilled water). Total protein content was determined using the Qubit protein assay kit as described by the manufacturer (Invitrogen, USA). Proteins were separated on a 4–12% gradient Mini-PROTEAN stain-free TGX gels (Bio-Rad, USA) and transferred to a polyvinylidene difluoride membrane. Protein transfer and content were investigated as described [42].

### 4.21. Nuclei Extraction and Flow Cytometry Analysis

Full root systems from the reported transgenic tomato composite plants were collected, and the subsequent nuclear isolation and staining was conducted as previously described [111] using the CyStain UV precise P kit (Sysmex, Norderstedt, Germany). Briefly, root material was homogenized in 200 µL of nuclei extraction buffer, followed by addition of 1 mL staining buffer. Resuspended nuclei were transferred to a polystyrene collection tube containing a mini strainer of 35 µm mesh. Flow cytometry assay was conducted on the final nuclei suspension using the CyFlow Space Analyzer with UV-laser excitation (Sysmex, Kobe, Japan). At least ten thousand nuclei were considered in each sample with the corresponding three technical replications. The endoreplication index represents the mean number of endoreplication cycles per root cell and was calculated as follows: % of 4C + 2 × % of 8C + 3 × % of 16C. Data are shown as average from three biological repeats and their SEM.

### 4.22. Statistical Analysis

Statistical analyses were performed on GraphPad Prism v9 software, and data was shown as mean ± SEM. Statistical significance was determined by Student’s *t*-test, one-way or two-way ANOVA, followed by multiple comparison α < 0.05. The detailed statistical information is shown in the Figure legends and “n” represents the number of samples used in one biological repeat.

## Data Availability

The original contributions presented in this study are included in the article/Supplementary Material. Further inquiries can be directed to the corresponding authors.

## Supplementary Materials

The following supporting information can be downloaded at: https://www.mdpi.com/article/10.3390/ijms262412178/s1.

## Abbreviations

The following abbreviations are used in this manuscript:

a%	arbuscule abundance in root fragments
A%	arbuscule abundance in the whole root
AM	arbuscular mycorrhiza
AMF	arbuscular mycorrhizal fungi
CDS	coding sequence
DAS	days after stratification
FL	full length
F%	mycorrhization frequency in the whole root
GFP	green fluorescent protein
m%	mycorrhization intensity in root fragments
M%	mycorrhization intensity in the whole root
NLS	nuclear localization signal
rBIFC	ratiometric bimolecular fluorescence complementation
RFP	red fluorescent protein
SAS10	SOMETHING ABOUT SILENCING 10
SP	signal peptide
SYM	symbiosis
wpi	weeks post inoculation
WGA	wheat germ agglutinin
WT	wild-type
YFP	yellow fluorescent protein
YST	yeast secretion trap
Y2H	yeast two-hybrid
