# Supplementary material for: The Nuclear Effector RIRG190 Interacts with SAS10 to Regulate Arbuscular Mycorrhizal Symbiosis
Source: Int J Mol Sci. 2025 Dec 18;26(24):12178. doi: 10.3390/ijms262412178 (PMC12733757; doi:10.3390/ijms262412178)
Supplement: Supplementary file 1 [file ijms-26-12178-s001.zip › ijms-4012739-supplementary.pdf]

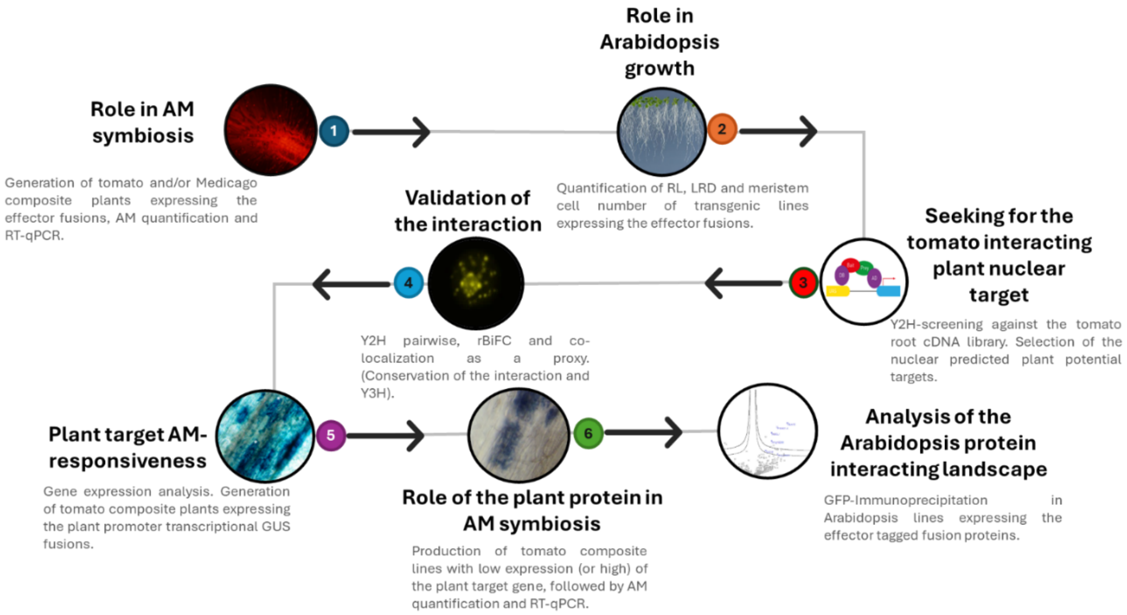

Figure S1. Schematic overview of the experiment workflow of this study.

A

MVAIKLIFVFLVITTTALVTAFNPQLMLKLVNDERKKVGASPLTLDSKLTSAAQKHTDFM
VKTDTLTHDDDAGSLGERIKAEGYNFSNAGENIAEGFGTNDEARVMKAWMGSSGHKA
NILNKAFTNLGVGFGGGKYWTQVFGKPLNSRKSKRFARKRLVRE

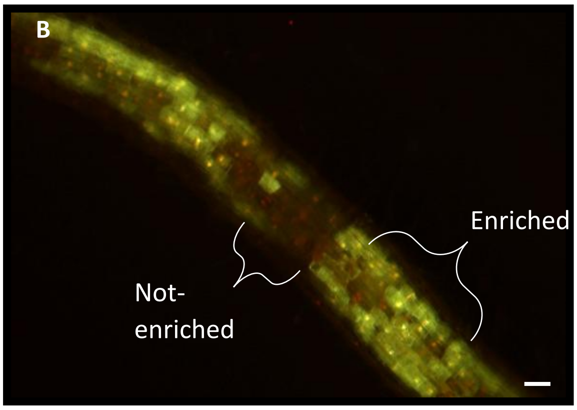

C

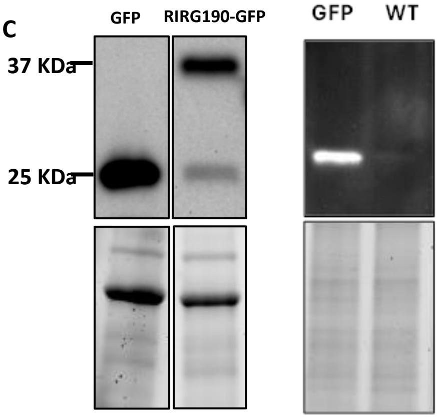

D

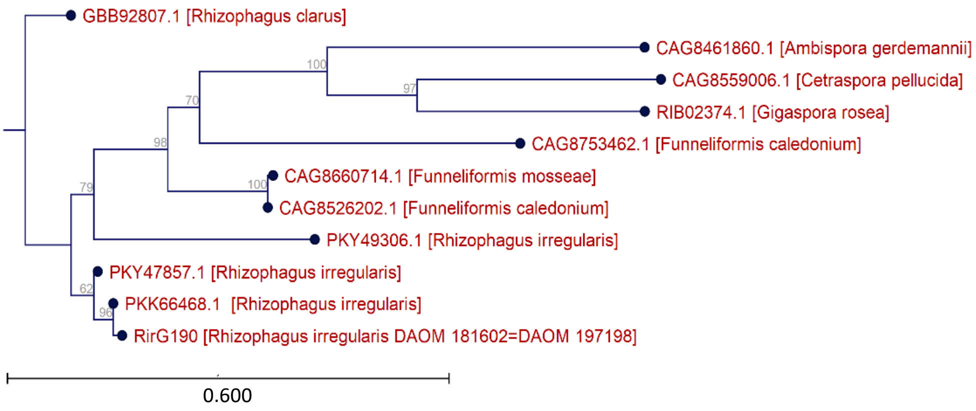

E

Sr.No.	AFDB accession	Description	Species	Residue Range	E-value	Sequence identity	Average pLDDT
1	AF-A0A2Z6QRV3-F1	SCP domain-containing protein	Rhizophagus clarus	1-160	2.70E-27	83.30%	89.69
2	AF-A0A397TML0-F1	CAP domain-containing protein	Glomus cerebriforme	1-175	3.44E-17	46.70%	85.56
3	AF-A0A4P9ZR19-F1	SCP domain-containing protein	Dimargaris cristalligena	1-392	4.92E-15	34.30%	55
4	AF-A0A1V6D2P8-F1	Cysteine-rich secretory protein family protein	Planctomycetes bacterium ADurb.Bin126	1-146	1.14E-14	39.10%	90.69
5	AF-A0A1Y1YUK7-F1	SCP-domain-containing protein	Basidiobolus meristosporus CBS 931.73	1-157	1.57E-14	34.20%	87.94
6	AF-A0A221NZQ2-F1	SCP domain-containing protein	Streptomyces pluripotens	1-255	1.78E-14	34.40%	70.62
7	AF-A0A3Q9FYI2-F1	CAP domain-containing protein	Streptomyces luteoverticillatus	1-167	1.90E-14	40.10%	87.31
8	AF-A0A7K3BKF6-F1	CAP domain-containing protein	Streptomyces sp. SID8377	1-256	1.90E-14	37.80%	72.12
9	AF-A0A2W6ZZF3-F1	SCP domain-containing protein	Leptolyngbya sp	1-174	2.16E-14	40.70%	86.12
10	AF-A0A1C5EA22-F1	Uncharacterized conserved protein YkwD, contains CAP (CSP/antigen 5/PR1) domain	Streptomyces sp. Ncost-T10-10d	1-179	2.99E-14	38.40%	84.88
11	AF-A0A1C4YG26-F1	Uncharacterized conserved protein YkwD, contains CAP (CSP/antigen 5/PR1) domain	Micromonospora matsumotoense	1-530	3.40E-14	40%	52.66
12	AF-A0A6N9UNG2-F1	CAP domain-containing protein	Streptomyces coelicoflavus	1-216	4.12E-14	39.50%	74.06
13	AF-A0A6G9YES9-F1	SCP domain-containing protein	Nocardia arthritidis	1-301	4.12E-14	38.80%	64.69
14	AF-A0A841FWB6-F1	Uncharacterized protein YkwD	Phytomonospora endophytica	1-237	4.40E-14	43%	76.06
15	AF-A0A7J5C507-F1	CAP domain-containing protein	Micromonospora sp. AMSO31t	1-349	5.00E-14	37.20%	61.72
16	AF-A0A420F2I2-F1	SCP domain-containing protein	Micromonospora globbae	1-569	5.00E-14	38.60%	51.25
17	AF-A0A849DMK0-F1	CAP domain-containing protein	Dactylosporangium sp.	1-278	5.34E-14	41.70%	67.94
18	AF-A0A4R4Q7V6-F1	CAP domain-containing protein	Micromonospora sp. 15K316	1-161	6.07E-14	41.60%	86.19
19	AF-A0A285QVR7-F1	Uncharacterized conserved protein YkwD, contains CAP (CSP/antigen 5/PR1) domain	Streptomyces sp. 1331.2	1-328	6.07E-14	39.70%	62.31
20	AF-A0A6H2NQ03-F1	CAP domain-containing protein	Leptolyngbya sp. LCM1.Bin17	1-182	6.47E-14	35.20%	80.75

Figure S2. RIRG190 is a nuclear-localized and AMF-conserved effector protein.

(A) Schematic representation and amino acid sequence of the RIRG190 effector protein. The effector displays a putative N-terminal SP domain of 21 amino acids (red) and a putative C-terminal NLS sequence of 15 amino acids (yellow). Representation was achieved using the illustrator for biological sequences (IBS) software.

(B) Fluorescent field picture of *SlPT4p:GFP RolDp:mRuby-NLS* mycorrhized tomato composite plants. Nuclear mRuby fluorescent signals (red) allow the screening of transformed roots, whereas GFP signals (green) specifically accumulate in arbuscule-containing cells. Bar, 50 μM.

(C) Western blot of GFP and RIRG190-GFP fusion proteins (left) and GFP and WT samples (right) to test GFP antibody specificity using anti-GFP monoclonal antibody. The molecular mass of fusion proteins is 27 kDa for GFP and 43 kDa for RIRG190-GFP. Visualization of the total amount of proteins after separation on stain-free polyacrylamide gels (lower panels) was achieved using the ChemiDoc imaging system (Bio-Rad, California).

(D) RIRG190-like effectors are conserved in different AMF species. The protein homology-based tree of RIRG190 effector-like proteins was generated using the neighbor-joining tree builder on CLC Workbench 8 software.

(E) Top 20 hits output of a structural similarity search of RIRG190 and AtPR1 with Foldseek in AlphaFold against the AFDB50 database.

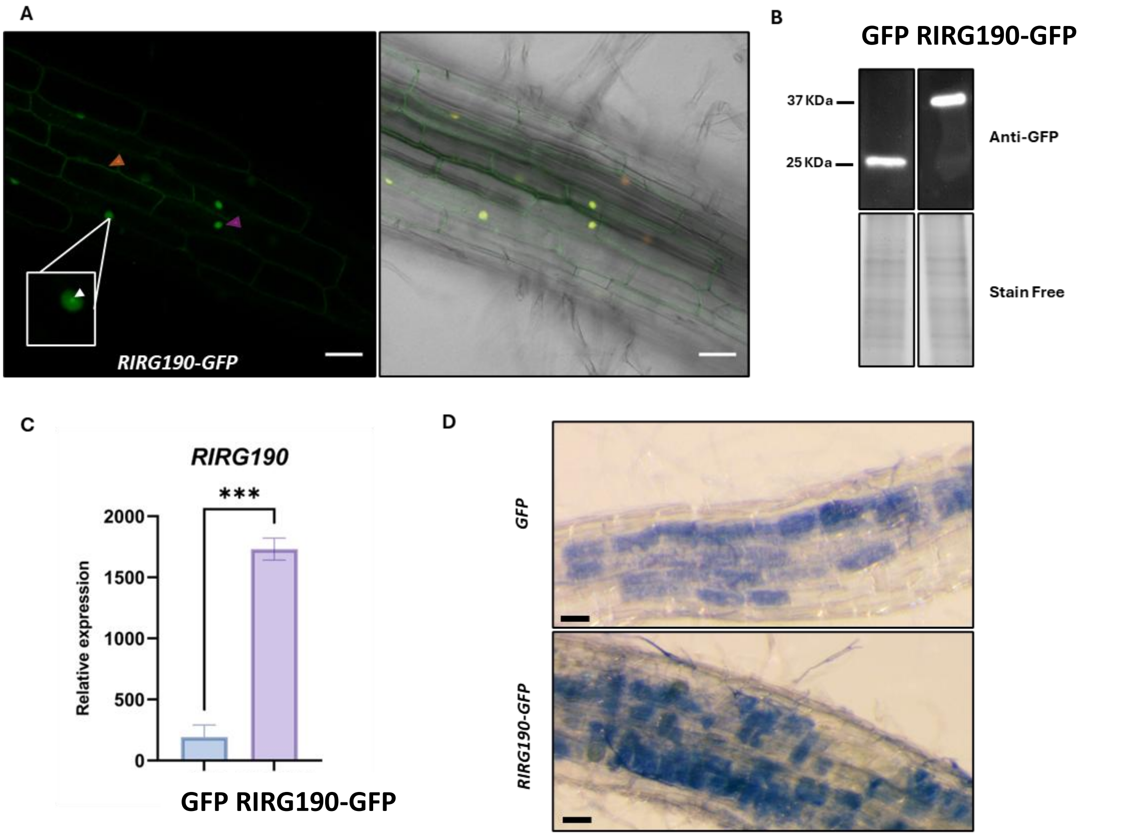

Figure S3. The ectopic expression of the nuclear-localized effector RIRG190 positively affects AM symbiosis.

(A) Confocal laser-scanning microscopy image of tomato roots overexpressing *RIRG190-GFP*. The left panel shows GFP signal accumulation in the plant cytoplasm (orange arrowhead), nucleus (purple arrowhead), and nucleolus (white arrowhead, inset). The right panel is a merge between the GFP channel, the bright field, and the constitutive *mRuby* NLS signal located at the backbone of the screening module, resulting in yellow fluorescent signals. Bars, 50 μM.

(B) GFP and RIRG190-GFP fusion proteins detected by Western blot using anti-GFP monoclonal antibody in tomato. The molecular mass of fusion proteins is 27 kDa for GFP and 43 kDa for RIRG190-GFP. Visualization of the total amount of proteins after separation on stain-free polyacrylamide gels (lower panels) was achieved using the ChemiDoc imaging system (Bio-Rad, California).

(C) *RIRG190-GFP* expression levels in mycorrhized tomato composite plants overexpressing *GFP* or *RIRG190-GFP*. Gene normalization was conducted using the tomato *SlEF1α* and *SlGAPDH* genes, with its relatively comparison to mycorrhized *GFP*

control lines at four wpi. Data are means ± SEM of three independent biological repeats (n = 9-12; ***, P < 0.001; Student's *t*-test).

(D) Bright-field visualization of ink-colored arbusculated cells in tomato. Bars, 50 μM.

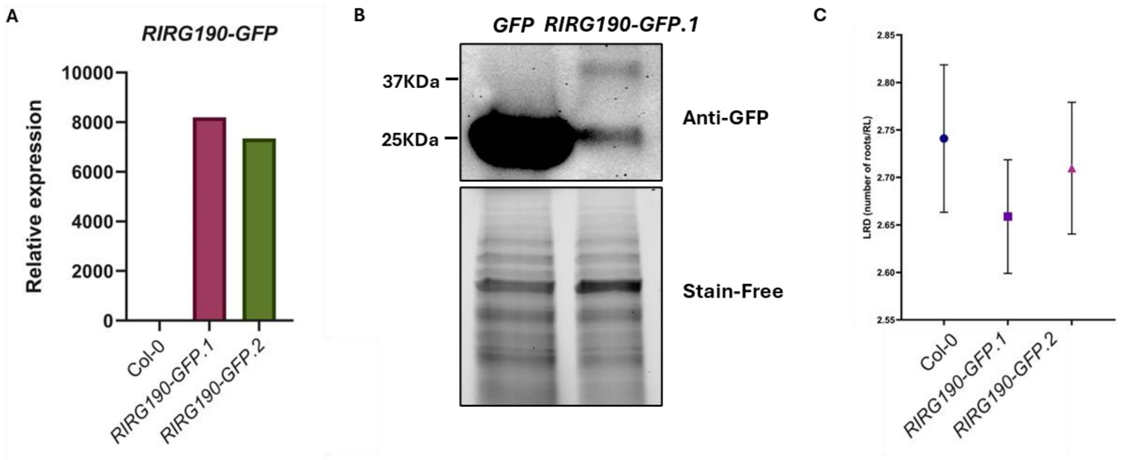

Figure S4. Arabidopsis *RIRG190-GFP* transgenic roots display increased *RIRG190-GFP* gene expression.

(A) *RIRG190* transcript levels in two-week-old Arabidopsis *RIRG190-GFP.1* and *RIRG190-GFP.2* transgenic roots. Transcript levels were normalized using *AtACTIN2* and *AtTUBULIN2* as housekeeping genes and relatively compared to wild-type Col-*0* roots. Expression was tested in a pool of 36 Arabidopsis RIRG190-GFP roots at 14 DAS.

(B) Corresponding GFP and RIRG190-GFP.1 protein fusions detected by Western blot using anti-GFP monoclonal antibody. Molecular weight of fusion proteins: GFP, 27 kDa; RIRG190, 43 kDa. Visualization of the total amount of proteins after separation on a stain-free polyacrylamide gel (lower panel) was achieved using the ChemiDoc imaging system (Bio-Rad, California).

(C) Lateral root density of Arabidopsis Col-*0*, RIRG190-GFP.1 and RIRG190-GFP.2 measured at 14 DAS. Values are means of two biological repeats (n = 32-66). No statistical differences were shown by a one-way ANOVA.

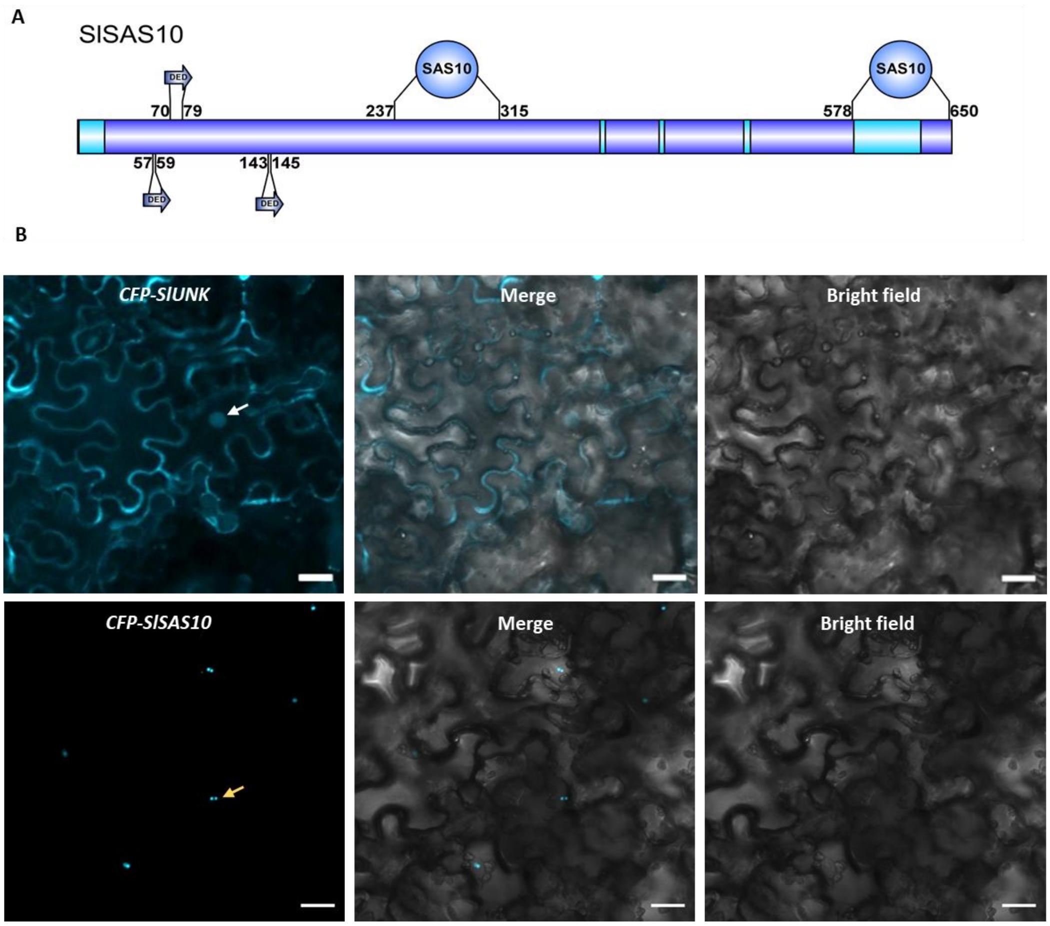

Figure S5. The nuclear-localized SlSAS10 protein contains motifs involved in chromatin regulation, ribosomal RNA processing, and cell homeostasis.

(A) Schematic representation of the tomato SlSAS10 protein sequence. NLS sites are indicated in light blue, SAS10 functional domains are represented by a circle, while N-terminal DED motifs are symbolized with an arrow.

(B) Subcellular localization of *CFP-SlSAS10* and *CFP-SlUNK* fusions in tobacco leaf epidermal cells. SlUNK fluorescence was detected in the cytoplasm and nuclei (white arrow) of leaf cells, while the CFP fluorescent signal of the *CFP-SlSAS10* fusion protein was observed in nuclear foci (yellow arrow). The merged image shows the combination of the bright field and the CFP field. Bars, 20 μM.

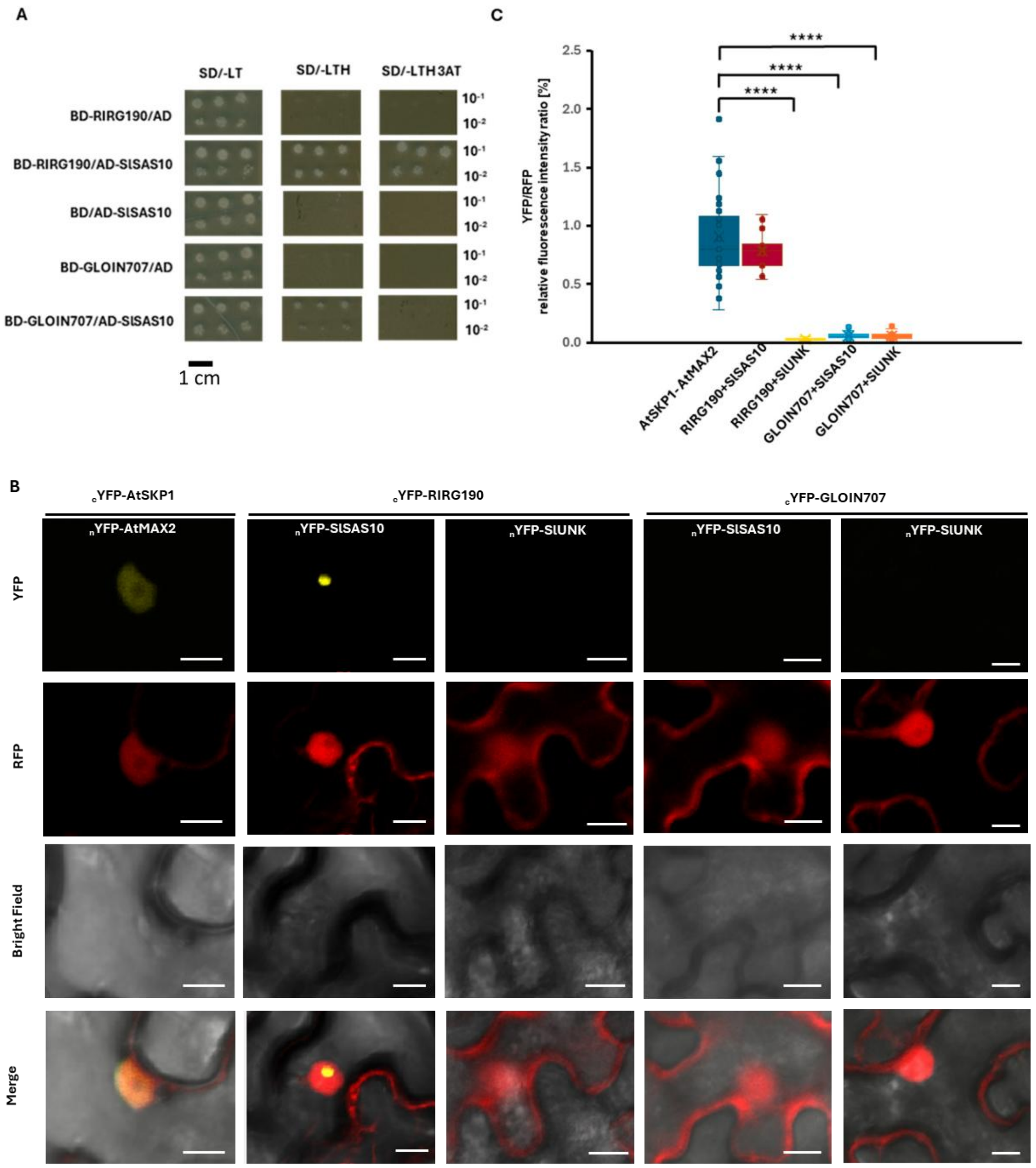

Figure S6. SlSAS10 does not interact with another nuclear-localized *R. irregularis* effector GLOIN707.

(A) Binary Y2H assays demonstrates the absence of interaction between GLOIN707 and SlSAS10. As a negative control, the tomato bait was cotransformed with the empty PGBKT7 vector (BD/+). Transformed PJ-69α cells were diluted and grown on SD/-LT control medium and SD/-LTH selective medium with or without 5 mM of 3-AT for 3 days at 30°C.

(B) rBiFC assay corroborates nuclear YFP reconstitution due to protein–protein interaction between RIRG190 and SlSAS10 N-terminal split YFP fusions. A nuclear YFP signal was also detected in the positive control AtSKP1-AtMAX2, whereas lack of YFP was observed for the RIRG190-SlUNK, GLOIN707-SlSAS10, and GLOIN707-SlUNK protein fusion pairs. The RFP fluorescent signal corresponds to the constitutively expressed control cassette (middle pictures). Bottom pictures show the overlay between YFP/RFP fluorescence. Three independent leaves were infiltrated and a total of 20-35 cells were analyzed. Bars, 10 μM.

(C) YFP/RFP relative fluorescent intensity analysis of the rBiFC protein pairs AtSKP1-AtMAX2 (positive control), RIRG190-SlSAS10, RIRG190-SlUNK, GLOIN707-SlSAS10, and GLOIN707-SlUNK; Data are shown as means of three biological replicates ± SEM (n = 20-35; ****, $P < 0.0001$; Student's *t*-test).

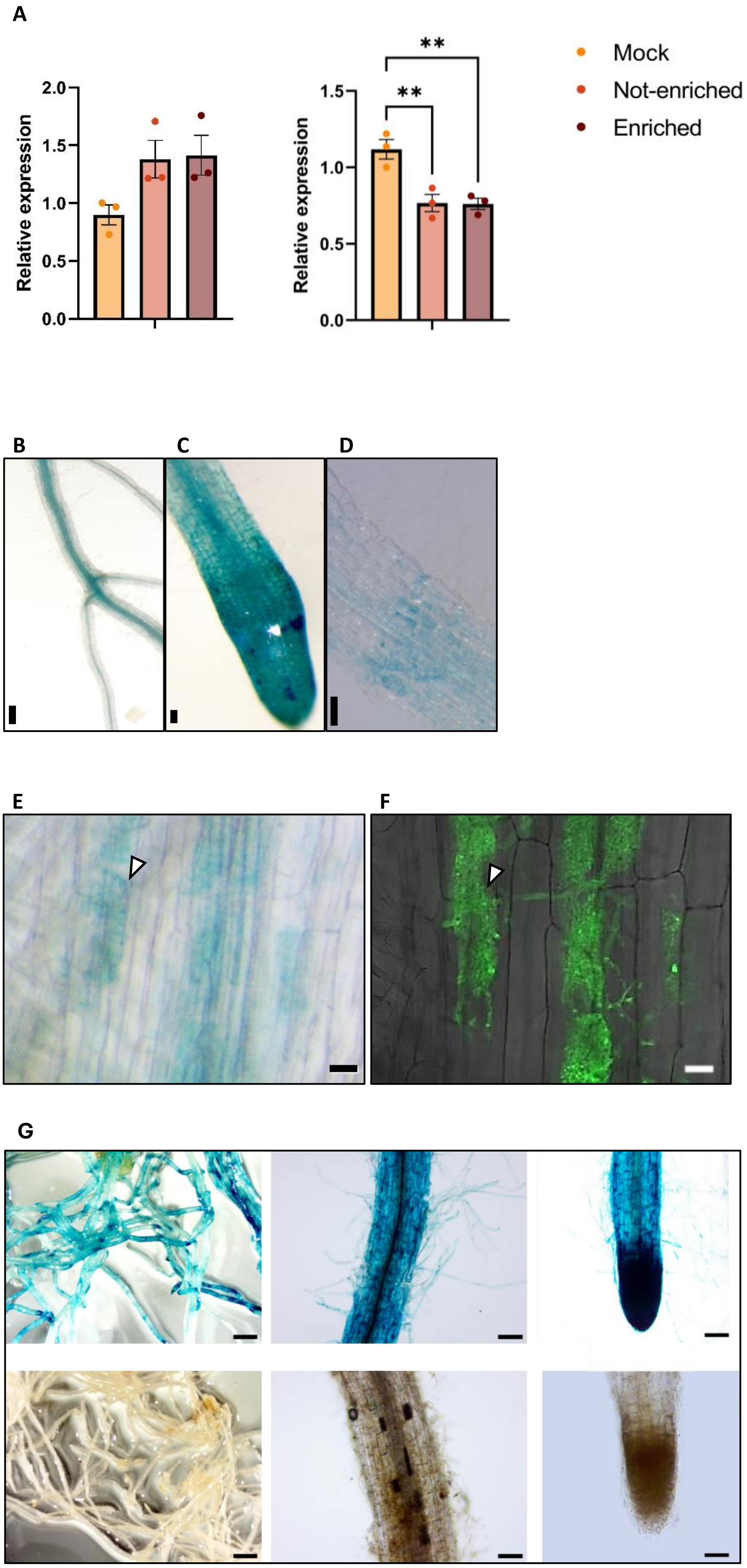
A
Mock
Not-enriched
Enriched
Relative expression
2.0
1.5
1.0
0.5
0.0
**
B
C
D
E
F
G

Figure S7. SlSAS10 might act at the arbuscular phase of AM symbiosis.

(A) *SlSAS10* expression levels at 2 wpi (left) and 4 wpi (right) in mock roots and mycorrhized root material enriched or not for arbuscule-containing cells. Values were normalized using *SlEF1α* and *SlGAPDH* housekeeping genes and relatively compared with mock-inoculated root samples. Data are represented as mean ± SEM of three independent biological repeats (n = 12; **, $P < 0.005$; One-way ANOVA followed by multiple comparisons (α<0.05)).

(B-D) *GUS* activity in 4-week-old tomato roots carrying the *SlSAS10p:GUS* construct in non-inoculated (B,C) and mycorrhized roots (D) at 4 wpi. Representative bright field pictures of six analyzed roots from three independent repeats. Bars, 500 μM (B), 100 μM (C), and 100 μM (D).

(E,F) Bright field and confocal images of inoculated tomato *SlSAS10p:GUS* roots counterstained with X-Gluc (E) and WGA (F). The arrowheads indicate the presence of arbuscules in the representative picture. Bars, 20 μM.

(G) GUS activity in 4-week-old roots of mycorrhized tomato transformed with the *35S:GUS* construct (upper panel) and the empty vector. Bars, 1 mm (left) and 0.1 mm (middle and right).

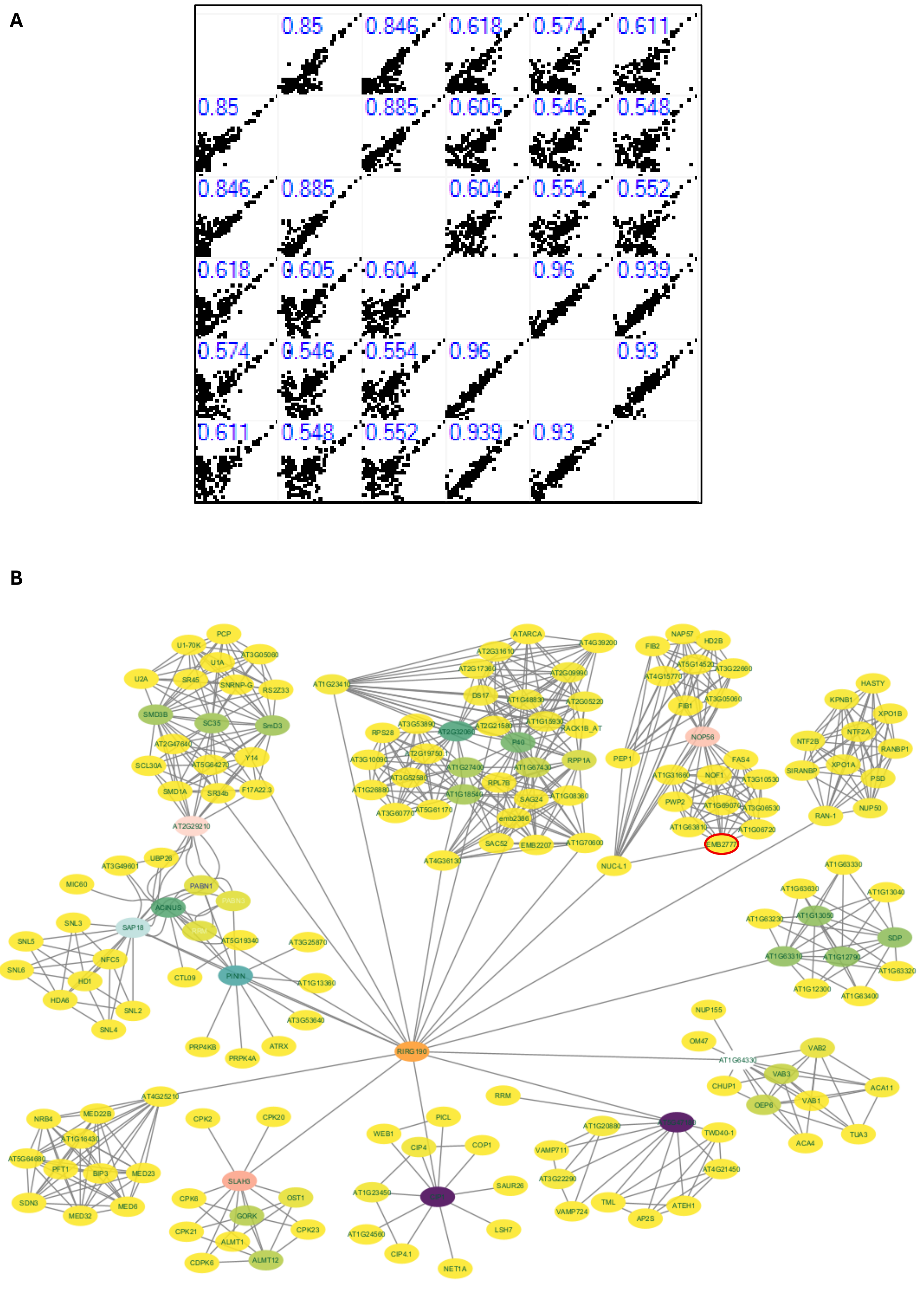

Figure S8. RIRG190 interacts with known SAS10-associated proteins.

(A) Pearson correlations among the different biological repeats in GFP-immunoprecipitated *GFP* and *RIRG190-GFP* samples.

(B) RIRG190 protein–protein interaction network visualization of closely associated proteins in Arabidopsis roots. The central node represents the effector RIRG190 connected by edges to the enriched preys found in the effector-enriched root protein extract. Darker nodes represent more influenced proteins in agreement to their connected nodes. AtSAS10/THAL, also known as EMB2777, is highlighted in red. Target proteins cluster in different groups, mainly involved in translation, rRNA processing, alterative splicing, chromatin remodeling, and trafficking.

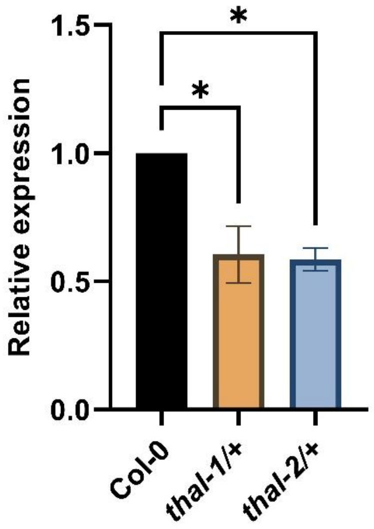

Figure S9. *AtSAS10* transcript downregulation in roots of Arabidopsis *thal*-/+ mutant lines. *AtACTIN2* and *AtTUBULIN2* housekeeping genes were used for normalization and values were compared against Col-*0* control lines. Data are shown as mean of two biological repeats with their SEM (n = 12; *, $P < 0.05$, One-way ANOVA followed by multiple comparison ($\alpha < 0.05$)).

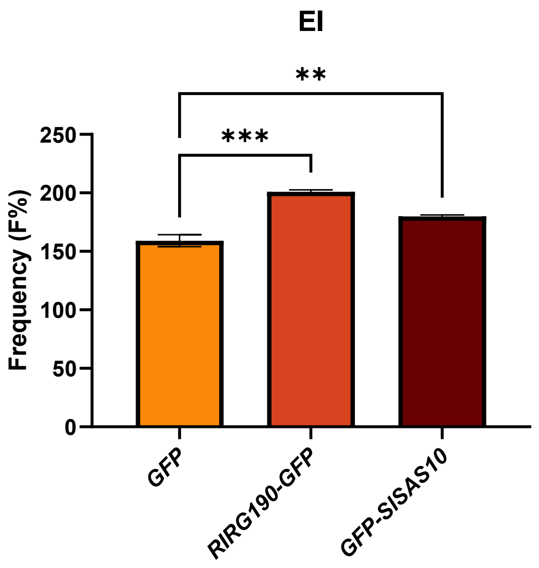

Figure S10. Impact of RIRG190 and SlSAS10 on the endocycle.

Endoreplication index (EI) of root cells of 4-week-old non-inoculated tomato composite plants overexpressing the fusion proteins *GFP*, *RIRG190-GFP*, and *GFP-SlSAS10*. Values are mean of three biological replicates ± SEM (n = 12; **, $P < 0.005$; ***, $P < 0.001$; One-way ANOVA).

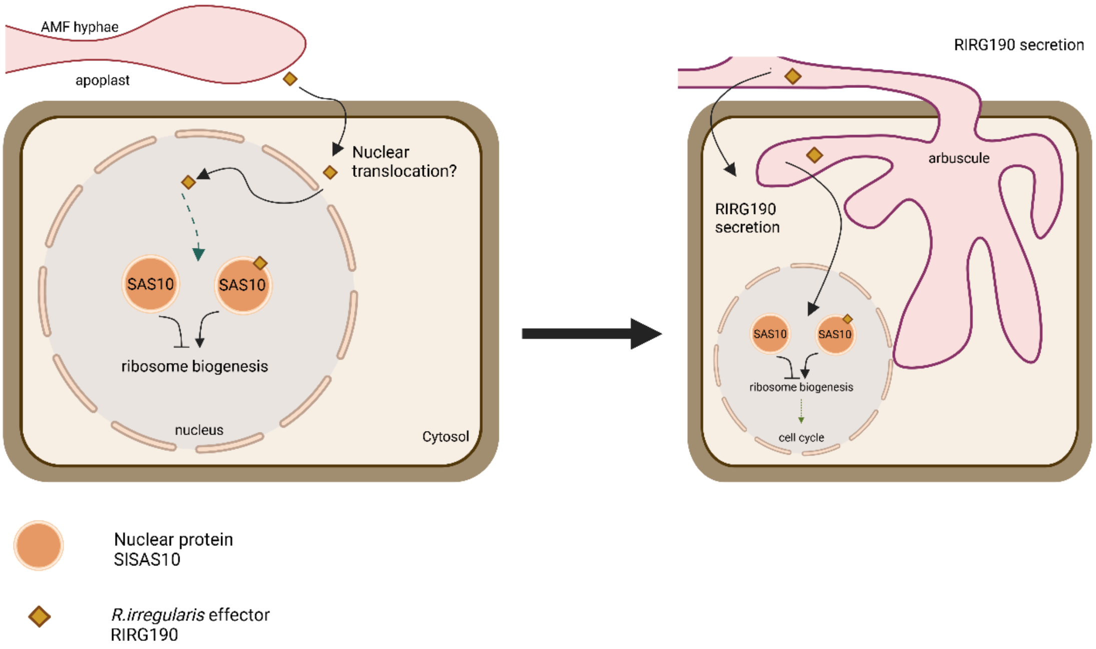

Figure S11. Hypothesized mode of action of RIRG190.

RIRG190 might be secreted by the fungus throughout the different stages of the symbiotic process, after which it is translocated to the cytosol of cortical cells, and partially compartmentalized in the plant nucleus of *S. lycopersicum* plants. Once in the nucleus, RIRG190 interacts with SlSAS10, probably leading to a derepression of SlSAS10 in the ribosomal biogenesis process [1]. This deregulation might lead to an activation of the cell cycle in cortical cells, which facilitates the arbuscule establishment.

Table S1. (A) List of primers used for cloning; (B) detailed composition of the vectors generated in this work; (C) RIRG190 effector *in silico* predictions; (D) RT-qPCR primer list; (E) Primers used to validate genomic T-DNA insertion; (F) MaxQuant parameters and Galaxy headers.

Table S1a. List of primers used for the generation of the different vectors in this work. ID, gene name; VECTOR, name of the vector referred to this research; NAME, vector's name; NUCLEOTIDE SEQUENCE, complementary DNA sequence. Blue color highlights the adaptor sequences, whereas red indicates START or STOP codons.

Table S1b. Detailed list of constructs used for each experimental technique in this work. EXPERIMENT, main experiment where vectors were used; CLONING, cloning approach used; NAME, vector's name; DESTINATION VECTOR, vector backbone; EXPRESSION VECTOR, detailed vector composition.

Table S1f. MaxQuant search parameters used in this work and Galaxy headers.

Dataset S1. (A) List of RIRG190-interacting candidates identified in the Y2H screening; (B) SAS10 protein alignment; (B) Differentially enriched proteins in Arabidopsis RIRG190-GFP immunoprecipitated fraction.

Reference

1. Y.-J. C. Chen, H.-J. Wang and G.-Y. Jauh, "Dual role of a SAS10/C1D family protein in ribosomal RNA gene expression and processing is essential for reproduction in *Arabidopsis thaliana*," *PLoS genetics*, vol. 12, no. 10, p. e1006408, 2016.
